# Insight into the reaction mechanism of lipoyl synthase: a QM/MM study

**DOI:** 10.1007/s00775-017-1522-8

**Published:** 2017-12-04

**Authors:** Geng Dong, Lili Cao, Ulf Ryde

**Affiliations:** Department of Theoretical Chemistry, Chemical Centre, Lund University, P.O. Box 124, 221 00 Lund, Sweden

**Keywords:** Lipoyl synthase, FeS cluster, Spin state, Reaction mechanism, QM/MM, Density functional theory

## Abstract

Lipoyl synthase (LipA) catalyses the final step of the biosynthesis of the lipoyl cofactor by insertion of two sulfur atoms at the C6 and C8 atoms of the protein-bound octanoyl substrate. In this reaction, two [4Fe4S] clusters and two molecules of *S*-adenosyl-l-methionine are used. One of the two FeS clusters is responsible for the generation of a powerful oxidant, the 5′-deoxyadenosyl radical (5′-dA^•^). The other (the auxiliary cluster) is the source of both sulfur atoms that are inserted into the substrate. In this paper, the spin state of the FeS clusters and the reaction mechanism is investigated by the combined quantum mechanical and molecular mechanics approach. The calculations show that the ground state of the two FeS clusters, both in the [4Fe4S]^2+^ oxidation state, is a singlet state with antiferromagnetically coupled high-spin Fe ions and that there is quite a large variation of the energies of the various broken-symmetry states, up to 40 kJ/mol. For the two S-insertion reactions, the highest energy barrier is found for the hydrogen-atom abstraction from the octanoyl substrate by 5′-dA^•^. The formation of 5′-dA^•^ is very facile for LipA, with an energy barrier of 6 kJ/mol for the first S-insertion reaction and without any barrier for the second S-insertion reaction. In addition, the first S ion attack on the C6 radical of octanoyl was found to take place directly by the transfer of the H6 from the substrate to 5′-dA^•^, whereas for the second S-insertion reaction, a C8 radical intermediate was formed with a rate-limiting barrier of 71 kJ/mol.

**Electronic supplementary material:**

The online version of this article (10.1007/s00775-017-1522-8) contains supplementary material, which is available to authorized users.

## Introduction

**Scheme 1:**
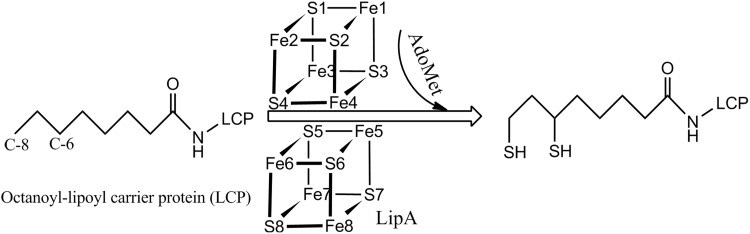
The sulfur-insertion reaction catalysed by LipA to form the lipoyl cofactor

Lipoic acid is a sulfur-containing cofactor that is essential for living organisms. It is employed in acyl-transfer reactions in several enzymes, including the pyruvate and α-ketoglutarate dehydrogenases in the citric acid cycle [1]. Lipoyl synthase (LipA) is a metalloenzyme that catalyses the final step in the biosynthesis of this cofactor by the insertion of two sulfur atoms at the C6 and C8 atoms of the octanoyl substrate, attached to the lipoyl carrier protein (Scheme 1) [2–5]. LipA belongs to the *S*-adenosyl-l-methionine (AdoMet) radical enzyme superfamily, which uses a [4Fe4S] cluster (termed the main cluster in this paper) to reductively cleave the C_5_′–S bond of AdoMet, generating l-methionine and a 5′-deoxyadenosyl radical (5′-dA^•^), which is a powerful oxidant and can be used to abstract a hydrogen atom from other molecules [6–9]. During the S-insertion reaction, two molecules of AdoMet are required (one for each sulfur insertion) to produce the lipoyl product [4]. An additional [4Fe4S] cluster (termed auxiliary FeS cluster) is also needed in the reaction, and it has recently been shown that it is the source of the inserted sulfur atoms [10, 11].

Crystallographic studies have shown that the two [4Fe4S] clusters are located in the active site of LipA [11, 12]. In the resting state (without octanoyl substrate and AdoMet binding to active site), the auxiliary [4Fe4S] cluster is bound to the protein with one Ser and three Cys residues, whereas three Cys resides and one solvent molecule coordinate to the other cluster. In the reactive state (when AdoMet and octanoyl groups bind to active site), the solvent ligand is replaced by the AdoMet group. During the first S-insertion reaction, the Ser residue dissociates from the auxiliary FeS cluster and one Fe ion is lost, whereas one of the sulfide ions is inserted into the substrate. This may facilitate the second S-insertion reaction [11–13].

[4Fe4S] clusters are versatile cofactors in enzymes, playing important roles in biological electron transfer, biosynthetic reactions, as iron sensors and as the sulfur source in biological radical reactions [14–16]. To understand the structure–function relationship of [4Fe4S] cluster, many studies have been performed, both experimental and theoretical [17–33]. Broken-symmetry density functional theory (BS-DFT) calculations of the [4Fe4S]^2+^ cluster (i.e., formally with two Fe^2+^, two Fe^3+^, and four S^2–^ ions) have shown that it consists of two high-spin ferromagnetically coupled [2Fe2S]^+^ (*S* = 9/2) subclusters, which together couple antiferromagnetically to the singlet state (*S* = 0) [24]. In each [2Fe2S]^+^ subcluster, the redox state of the two Fe ions is identical (Fe^2.5+^). In addition, a recent S K-edge X-ray absorption spectroscopy study of [4Fe4S] clusters bound to AdoMet in pyruvate formate-lyase activating enzyme has suggested that there is a back-bonding interaction between the [4Fe4S] cluster and the C–S σ* orbital of AdoMet [22]. This is supported by DFT calculations, which indicate that such an interaction facilitates the electron transfer between the FeS cluster and AdoMet.

**Scheme 2:**
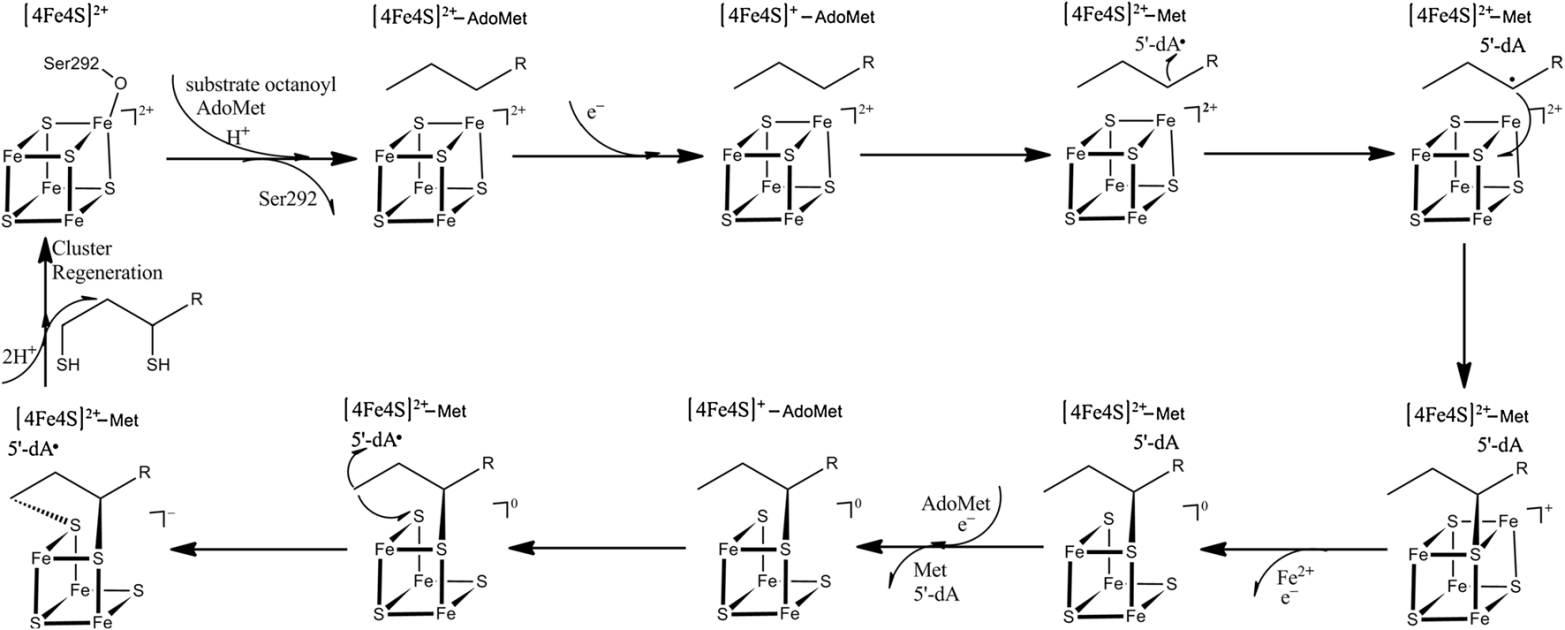
The proposed reaction mechanism for LipA, showing the main FeS cluster on the top and the auxiliary FeS cluster in full detail at the bottom

A reaction mechanism of LipA has been proposed based on the crystal structures (Scheme 2) [11, 12]. The redox states of the two FeS clusters in the resting state are both [4Fe4S]^2+^. The reaction starts with the binding of AdoMet and octanoyl substrate to the active site, the former coordinating directly to the main FeS cluster by replacing the water ligand. During this process, Ser292 is protonated and dissociates from the auxiliary FeS cluster. Next, the main FeS cluster is reduced by one electron, giving the [4Fe4S]^+^–AdoMet state. This triggers the cleavage of the C_5_′–S bond in AdoMet to generate [4Fe4S]^2+^–Met and the powerful oxidant 5′-dA^•^. The latter can abstract a hydrogen atom (H·) from the C6 atom of the octanoyl substrate. Then, the C6 radical will be attacked by a nearby S^2−^ ion of auxiliary FeS cluster in the first S-insertion reaction. This will reduce the auxiliary FeS cluster to the [4Fe4S]^+^ state. The first half-reaction is completed by the dissociation of one Fe^2+^ ion from the auxiliary cluster and a one-electron oxidation, giving the [3Fe3S]^0^ state with one Fe^3+^ and two Fe^2+^ ions.

The second half-reaction starts with the dissociation of the Met and 5′-dA products and the binding of a new AdoMet molecule. Again, the reaction is started by the reduction of the main FeS cluster to the [4Fe4S]^+^ state, which triggers the cleavage of the C5′–S bond in AdoMet, leading to the formation of 5′-dA^•^. Next, 5′-dA^•^ abstracts one hydrogen atom from C8 of the octanoyl substrate, generating a C8 radical, which will be attacked by another S^2−^ ion of the auxiliary FeS cluster. This produces the final product lipoyl and the auxiliary FeS cluster in the fully reduced [3Fe3S]^–^ state. Finally, the product can be released by binding two protons and a new auxiliary [4Fe4S] cluster needs to be regenerated before the enzyme is functional again.

In this paper, we have investigated this reaction mechanism of LipA with combined quantum mechanical and molecular mechanics (QM/MM) calculations. This allows us to understand how the redox and spin states of the FeS clusters change during the reaction mechanism, as well as how the sulfur ions of the auxiliary FeS cluster attack the octanoyl substrate.

## Methods

### The protein

All calculations were based on the 1.64 and 1.86-Å crystal structures of lipoyl synthase from *Mycobacterium tuberculosis* (PDB codes 5EXJ and 5EXK); the former is in the resting state and the latter is in an intermediate state, after the formation of the first C–S bond [11]. The crystal structure of resting state is a monomer, in which one molecule of dithiothreitol (DTT) binds to the main [4Fe4S] cluster. It was replaced by a water molecule in our calculations. In the crystal of the intermediate state, there are six independent proteins in the asymmetric unit, and we used chains A and B in our calculations. Chain B is a short octapeptide containing the octanoyl substrate, modelling the lipoyl carrier protein.

The setup of the enzyme was the same as in our previous calculations [34, 35]. The protonation states of all the residues were determined using PROPKA [36] and by a detailed study of the solvent accessibility, the hydrogen-bond pattern, and the possible formation of ionic pairs. They were the same in both the resting and the intermediate states. All Arg, Lys, Asp, and Glu residues were assumed to be charged. Cysteine ligands coordinating to metals were deprotonated. Among the His residues, His52 and 256 were assumed to be protonated on the NE2 atom, His179 was assumed be protonated on ND1, whereas His257 was modelled as doubly protonated. In addition, Ser292 in the resting state was deprotonated, because it binds to an Fe ion of auxiliary FeS cluster.

The protein was protonated and solvated with water molecules forming a sphere with a radius of 35 Å around the geometric centre using the leap module of the Amber software package (~ 15 000 atoms in total) [37]. The added protons and water molecules were optimised by a 120-ps simulated annealing calculation, followed by a minimisation, keeping the other atoms fixed at their crystal-structure positions. The protein was described by the Amber ff14SB force field [38] and water molecules with the TIP3P model [39]. No bonds were defined between the metal ions and their ligands (because they were kept fixed in the simulations).

### QM calculations

**Fig. 1:**
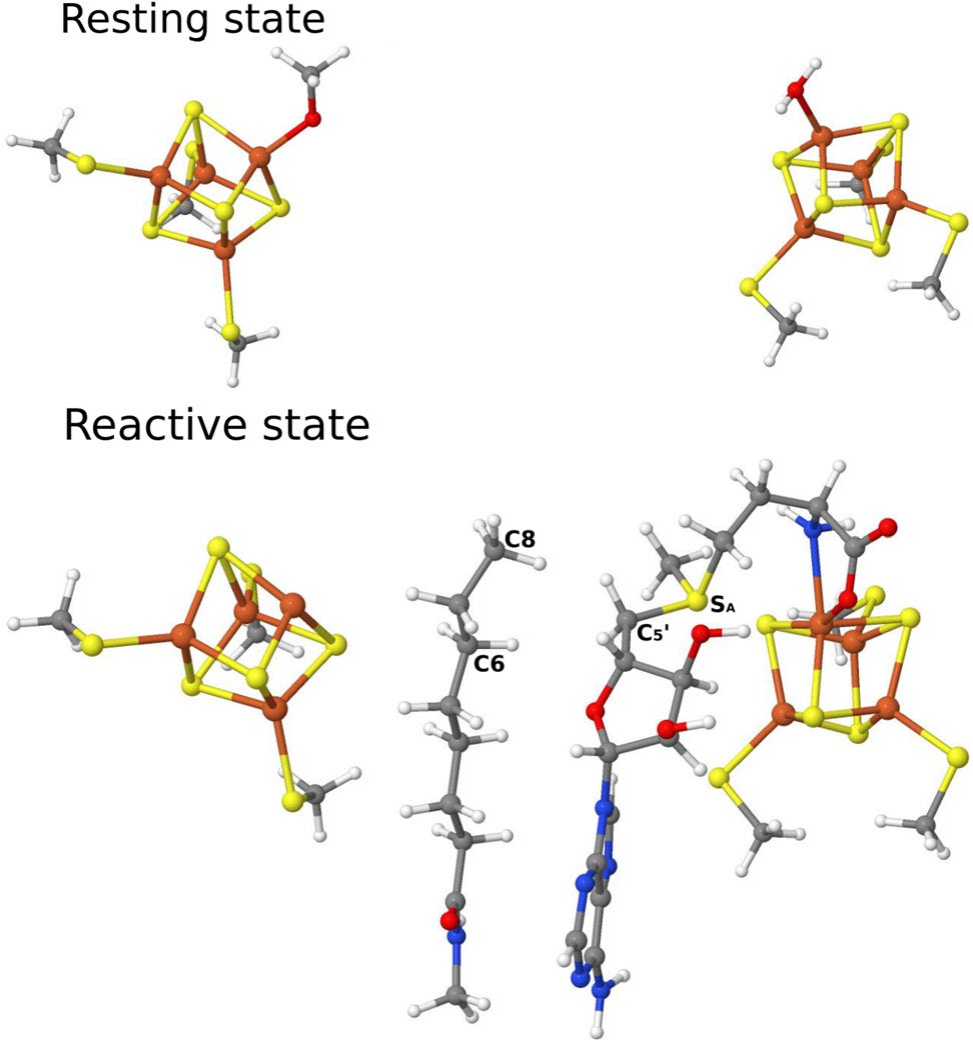
QM regions employed in the QM/MM calculations of the resting and reactive states, respectively. The auxiliary FeS cluster is shown to the left

All QM calculations were performed with the Turbomole 7.1 software [40]. Two DFT methods, TPSS [41] and B3LYP [42–44], and two different basis sets were used, def2-SV(P) [45] and def2-TZVP [46]. The calculations were sped up by expanding the Coulomb interactions in an auxiliary basis set, the resolution-of-identity (RI) approximation [47, 48]. All the calculations used a QM system consisting of the two [4Fe4S] clusters with all the first-sphere ligands (shown in Fig. 1). The Cys and Ser residues in the QM region were truncated by converting the CA atom to a hydrogen atom. In the intermediate state, the QM system was extended by the full AdoMet molecule, as well as the octanoyl substrate, which were modelled as shown in Fig. 1. To find all the possible BS states, two approaches were employed, viz. the fragment method developed by Szilagyi and Winslow, as implemented in the local program makebs [49], and a rapid generation of various BS states based on swapping the Fe ion coordinates [50].

### QM/MM calculations

The QM/MM calculations were performed with the ComQum software [51, 52]. In this approach, the protein and solvent are split into two subsystems: System 1 (the QM region) was relaxed by QM methods. It contained the same atoms as the vacuum QM calculations (shown in Fig. 1). System 2 contained the remaining part of the protein and the solvent. It was kept fixed at the original (crystallographic) positions.

In the QM calculations, system 1 was represented by a wavefunction, whereas all the other atoms were represented by an array of partial point charges, one for each atom, taken from MM libraries. Thereby, the polarisation of QM region by the surroundings is included in a self-consistent manner (electrostatic embedding). When there is a bond between systems 1 and 2 (a junction), the hydrogen link-atom approach was employed: The QM region was capped with hydrogen atoms (hydrogen link atoms, HL), the position of which are linearly related to the corresponding atom (carbon link atoms, CL) in the full system [52, 53]. All atoms were included in the point-charge model, except the CL atoms [54].

The total QM/MM energy in ComQum was calculated as [51, 52]

1 $$E_{{{\text{QM}}/{\text{MM}}}} = E_{{{\text{QM}}1 + {\text{ptch}}2}}^{\text{HL}} + E_{{{\text{MM}}12,q_{1} = 0}}^{\text{CL}} - E_{{{\text{MM}}1,q_{1} = 0}}^{\text{HL}} ,$$

where $E_{{{\text{QM}}1 + {\text{ptch}}2}}^{\text{HL}}$ is the QM energy of the QM region truncated by HL atoms and embedded in the set of point charges modelling system 2 (but excluding the self-energy of the point charges). $E_{{{\text{MM}}1,q_{1} = 0}}^{\text{HL}}$ is the MM energy of the QM system, still truncated by HL atoms, but without any electrostatic interactions. Finally, $E_{{{\text{MM}}12,q_{1} = 0}}^{\text{CL}}$ is the classical energy of all atoms in the system with CL atoms and with the charges of the QM system set to zero (to avoid double counting of the electrostatic interactions). By this approach, which is similar to the one used in the ONIOM method [55], errors caused by the truncation of the QM system should cancel.

The geometry optimisations were continued until the energy change between two iterations was less than 2.6 J/mol (10^−6^ a.u.) and the maximum norm of the Cartesian gradients was below 10^−3^ a.u. The QM calculations were carried out using the Turbomole 7.1 software [40]. Geometry optimisation was performed using the TPSS [41] functional in combination with def2-SV(P) [45] basis set, including the empirical DFT-D3 dispersion correction in Turbomole [56]. The MM calculations were performed with the Amber software, using the Amber ff14SB force field [38].

Reported energies are QM/MM energies obtained at the TPSS/def2-SV(P) level, including dispersion and the MM correction ($E_{\text{QM/MM}}^{\text{SV(P)}}$). This energy was then extrapolated to the B3LYP/def2-TZVP level using QM calculations with the same QM region and including the point-charge model:

2 $$E_{\text{tot}} = E_{\text{QM/MM}}^{\text{SV(P)}} + E_{\text{TPSS}}^{\text{TZVP}} + E_{\text{B3LYP}}^{\text{SV(P)}} - 2 E_{\text{TPSS}}^{\text{SV(P)}}$$

## Results and discussion

In this paper, we have studied the reaction mechanism of LipA. We first discuss the spin state of the two [4Fe4S]^2+^ cluster in the active site with and without the octanoyl substrate bound. Then, the determined ground state of FeS clusters will be used to investigate the mechanism of the two S-insertion reactions.

### The spin state of FeS cluster

The properties of [4Fe4S]^2+^ clusters have been studied extensively [17–33]. However, they can be affected by environment, e.g., different ligands binding to Fe ion or the protein surrounding. In this work, the QM/MM approach was employed, taking into account the surrounding protein and solvent. In the resting state, both clusters are in the [4Fe4S]^2+^ oxidation state, i.e., each with two reduced Fe^2+^ and two oxidised Fe^3+^ ions. This gives a total of 4 × (4 + 5) = 36 unpaired spins in the high-spin state, which can be combined in many different ways. We tried several spin states and in agreement with the previous studies, our results indicate that the lowest energy is obtained if both clusters are in the singlet state with antiferromagnetically coupled high-spin Fe ions (i.e., two ions with a surplus of spin up and two ions with a surplus of spin down). Mulliken spin analysis indicated that each Fe ion had approximately four unpaired spins each, either positive or negative.

**Fig. 2:**
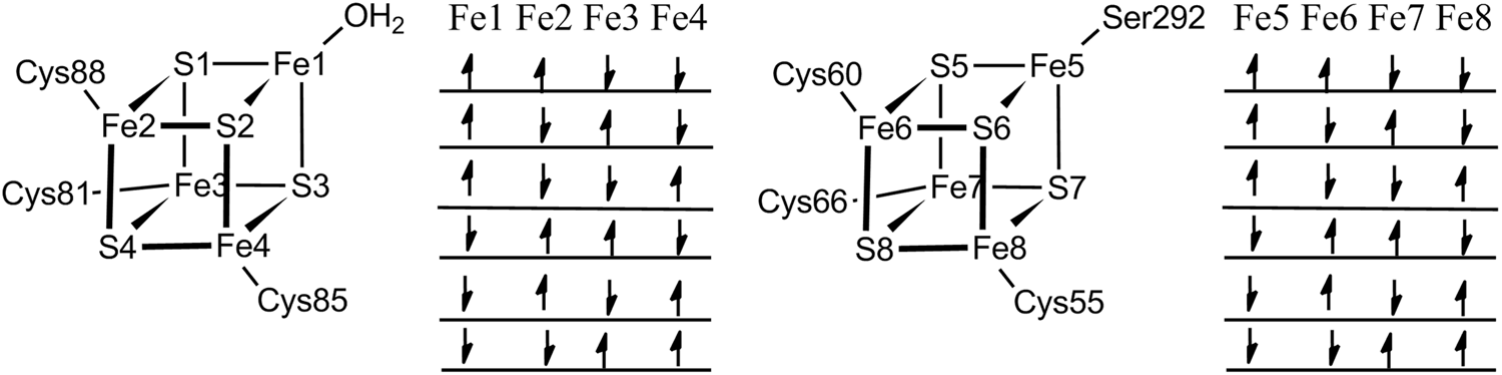
Six possible spin states of each of the [4Fe4S]^2+^ clusters in the resting state of LipA (main cluster to the left, auxiliary cluster to the right). The arrows indicate whether the Fe ions have a surplus of spin up or down

In each cluster, two Fe ions with spin up (and two with spin down) can be selected in six different ways, as shown in Fig. 2. Since the two clusters are quite far away (~ 12 Å), we first varied the spin state of the auxiliary cluster, keeping that of the main cluster constant. Then, we determined the most stable state for the main cluster. For each state, the geometries were optimised with QM(TPSS/def2-SV(P))/MM approach. The resulting energies are collected in Table 1. It can be seen that the spin state [↓↑↑↓] is predicted to be most stable for the auxiliary cluster and that the energies vary by up to 9 kJ/mol for the resting state when the spin of main FeS cluster is kept fixed. Then, we fixed the spin state of auxiliary cluster at the most stable state and varied the spin of the main cluster. As can be seen in Table 1, this gave a slightly larger variation in the energies by up to 13 kJ/mol. Interestingly, three states had nearly the same energy (within 2 kJ/mol), so it is hard to pinpoint which of these three states is the ground state.

**Table 1:** Energies for various spin state of two FeS clusters in the resting and reactive states of LipA (in kJ/mol). The arrows show the spin states of Fe ions, in the order Fe1–Fe8, according to the numbering in Fig. 2

Fixed spin	Spin state	Energy (kJ/mol)	
		Resting state	Reactive state
Main cluster	↑↓↑↓ ↓↑↓↑	0	0
	↑↓↑↓ ↑↓↓↑	− 4.7	23.7
	↑↓↑↓ ↑↑↓↓	2.7	37.4
	↑↓↑↓ ↓↓↑↑	1.9	0.4
	↑↓↑↓ ↓↑↑↓	− 5.9	− 2.1
	↑↓↑↓ ↑↓↑↓	− 0.9	21.2
Auxiliary cluster	↓↑↑↓ ↓↑↑↓	− 19.3	1.8
	↓↓↑↑ ↓↑↑↓	− 18.9	–
	↑↑↓↓ ↓↑↑↓	− 17.7	− 9.1
	↑↓↓↑ ↓↑↑↓	− 14.4	0.7
	↓↑↓↑ ↓↑↑↓	− 7.6	5.1

Next, we examined the spin states for the reactive state, in which AdoMet and the octanoyl substrate have bound to the active site of LipA, and Ser292 and the water ligand have dissociated from the FeS clusters. In addition, the main cluster was reduced by one electron to the [4Fe4S]^+^ state. This species is ready for the cleavage of C_5_′–S_A_ bond in AdoMet. As shown in Fig. 1, the AdoMet group binds to the Fe ion that was bound by a water molecule in resting state. To compare with the resting state, calculations with the same spins states as for the resting state were performed. The energies are also shown in Table 1. As for the resting state, the [↓↑↑↓] state is predicted to be most stable for the auxiliary cluster. However, the energies vary much more than for the resting state, by up to 40 kJ/mol. In particular, it can be seen that all states with Fe5 spin up are high in energy. In the reactive state, this ion is coordinated by only three S^2−^ ions, whereas the other Fe ions in this cluster also are bound to a Cys residue and it is also the ion that dissociates during the first S-insertion reaction. When this ion has a negative spin, the spin density is also lower (in absolute terms, e.g., − 3.4 with B3LYP) than for all the other Fe ions (3.6–3.8). However, when it has positive spin, it does no longer have a lower spin than the other Fe ions. The effect is especially pronounced at the B3LYP level of theory and the energy difference between the states with positive or negative spin on Fe5 increases by 14–22 kJ/mol with this functional.

The spin states of main cluster were also examined in the same way with the most stable spin state of auxiliary cluster. From Table 1, it can be seen that the variation in the energies is similar to that of the resting state, up to 14 kJ/mol. For the reactive state, the [↑↑↓↓ ↓↑↑↓] spin state gives the lowest energy and it is 7 kJ/mol lower than any other state. However, we could not locate the species with the [↓↓↑↑ ↓↑↑↓] spin state, because the S–C bond of AdoMet was cleaved during geometry optimisation, even if we first fixed the distance. To estimate the energy of this state, we performed a calculation for [↑↓↑↓ ↓↑↑↓] state, using the second lowest spin state of the auxiliary cluster. It was 11 kJ/mol higher than the [↑↑↓↓ ↓↑↑↓] state. Since the [↓↑↓↑] state is 2 kJ/mol higher than [↓↑↑↓] state in auxiliary cluster, it can be estimated that [↓↓↑↑ ↓↑↑↓] state is roughly 8 kJ/mol less stable than [↑↑↓↓ ↓↑↑↓] state. Therefore, we can conclude that the [↑↑↓↓ ↓↑↑↓] state is the most stable state in the reactive state and among the three lowest states for the resting state. Therefore, we used this state for the study of the reaction mechanism and we will call it the reactive spin state (RSS) throughout this paper.

The Fe–S^2−^ bond lengths in the two FeS clusters with the RSS spin state in resting and reactive states are shown in Table 2. For the resting state, the three Fe1–S bond lengths in the main cluster are 2.27–2.35 Å, which are slightly shorter than the corresponding bond lengths of Fe2–S (2.30–2.38 Å), Fe3–S (2.28–2.37 Å) and Fe4–S (2.27–2.36 Å). This is caused by the fact that Fe1 binds a water molecule, whereas the others are bound to Cys residues. On the other hand, the Fe1–S bonds (2.30–2.43 Å) became longer when AdoMet binds to Fe1 in the reactive state with both the carboxylate O atom. For the auxiliary cluster in the resting state, Fe5 binds to a Ser residue and consequently, the Fe5–S bonds are slightly longer than the other Fe–S bonds. On the other hand, for the reactive state, the Fe5–S bonds in the auxiliary cluster become shortest (2.27–2.28 Å), because no other groups bind to Fe5 except the three S^2−^ ions.

**Table 2:** Fe–ligand distances (in Å) of FeS clusters in the resting and reactive states with the RSS spin state

Main FeS cluster			Auxiliary FeS cluster		
Bond	Resting state	Reactive state	Bond	Resting state	Reactive state
Fe1–S1	2.32	2.43	Fe5–S5	2.31	2.28
Fe1–S2	2.35	2.37	Fe5–S6	2.37	2.27
Fe1–S3	2.27	2.30	Fe5–S7	2.35	2.27
Fe1–O_W_^a^	2.12	–	Fe5–O_Ser_	1.86	–
Fe1–O_A_^b^	–	2.18			
Fe1–N_A_^c^	–	2.28			
Fe2–S1	2.35	2.33	Fe6–S5	2.35	2.36
Fe2–S2	2.38	2.34	Fe6–S6	2.27	2.31
Fe2–S4	2.30	2.27	Fe6–S8	2.34	2.34
Fe2–S_Cys_	2.28	2.32	Fe6–S_Cys_	2.28	2.29
Fe3–S1	2.28	2.30	Fe7–S5	2.36	2.37
Fe3–S3	2.34	2.35	Fe7–S7	2.25	2.32
Fe3–S4	2.37	2.34	Fe7–S8	2.37	2.37
Fe3–S_Cys_	2.28	2.29	Fe7–S_Cys_	2.27	2.26
Fe4–S2	2.27	2.30	Fe8–S6	2.36	2.35
Fe4–S3	2.34	2.36	Fe8–S7	2.34	2.36
Fe4–S4	2.36	2.33	Fe8–S8	2.25	2.24
Fe4–S_Cys_	2.33	2.29	Fe8–S_Cys_	2.29	2.28

^a^ O_w_ is the oxygen atom in water

^b^ O_A_ is one of the carboxylate oxygen atoms in AdoMet

^c^ N_A_ is the amine nitrogen atom in AdoMet

### The mechanism of LipA

In the reactive state (RS_1_), AdoMet and the octanoyl substrate are bound to the active site of LipA and the auxiliary FeS cluster has been reduced. For convenience, we describe the states of the FeS clusters as [2Fe^2+^2Fe^3+^4S^2−^] and [3Fe^2+^Fe^3+^4S^2−^], even if the redox states of individual Fe ions cannot be discerned.

#### The first S-insertion reaction

**Fig. 3:**
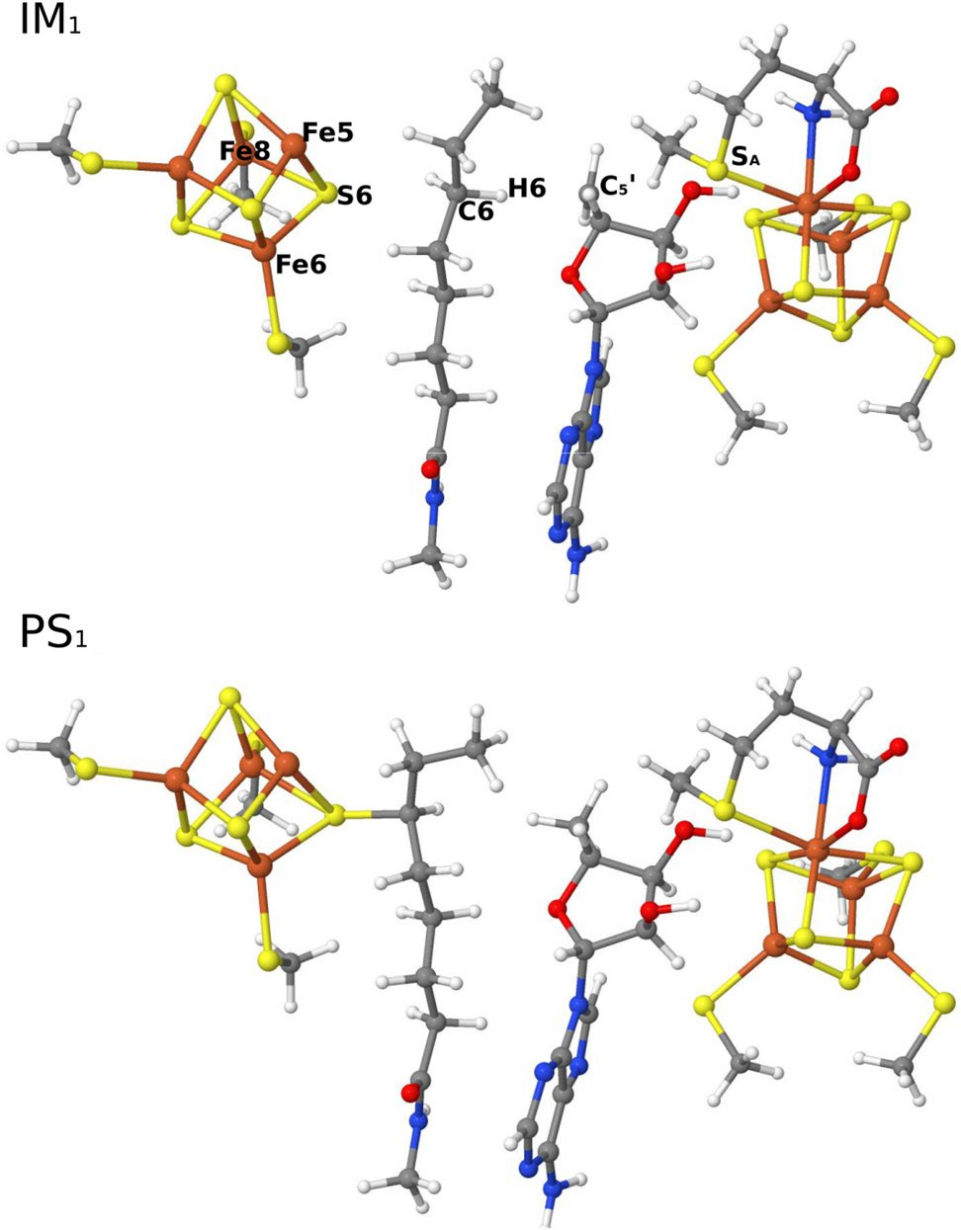
Structures of the IM_1_ intermediate and the PS_1_ product of the first S-insertion reaction

The reduction of the main FeS cluster is beneficial for the transfer of one electron to AdoMet and cleavage of the C_5_′–S_A_ bond of AdoMet. Our calculations indicate that cleavage of the C_5_′–S_A_ bond is heterolytic and very facile, with an energy barrier of only 6 kJ/mol and an exothermicity of 42 kJ/mol. During the reaction, one electron transferred from the main FeS cluster to AdoMet, producing 5′-dA^•^ and both FeS clusters in the [2Fe^2+^2Fe^3+^4S^2−^] oxidation state (i.e., the same as the resting state). The resulting IM_1_ state (the subscript 1 indicates that it belongs to the first S-insertion reaction) is shown in Fig. 3. It has a C_5_′–S_A_ distance of 3.5 Å, whereas the C_5_′–H6 distance is 2.2 Å. The *S*_A_ atom coordinates weakly to Fe1 with a distance of 2.8 Å.

Next, the powerful oxidant 5′-dA^•^ abstracts H6 from the C6 atom of the octanoyl substrate. The product of this reaction (PS_1_) is also shown in Fig. 3. The energy barrier was 37 kJ/mol. Interestingly, the substrate radical (C6 radical, Scheme 2) was not an intermediate state. Instead, C6 binds directly to S6 of the auxiliary FeS cluster, so that the first S-insertion product is directly generated. This PS_1_ state was 184 kJ/mol lower in energy than the reactive state. The S6–Fe5, S6–Fe6, and S6–Fe8 bond distances are 2.41, 2.40, and 2.56 Å in PS_1_ state, which are longer than in IM_1_ state (2.27, 2.28, and 2.34 Å, respectively), indicating that the formation of the S6–C6 bond weakens the S6–Fe bonds. In addition, during the S-insertion reaction, one electron is transferred from substrate to the main FeS cluster, leaving it in the reduced [3Fe^2+^Fe^3+^4S^2−^] state.

#### The second S-insertion reaction

**Fig. 4:**
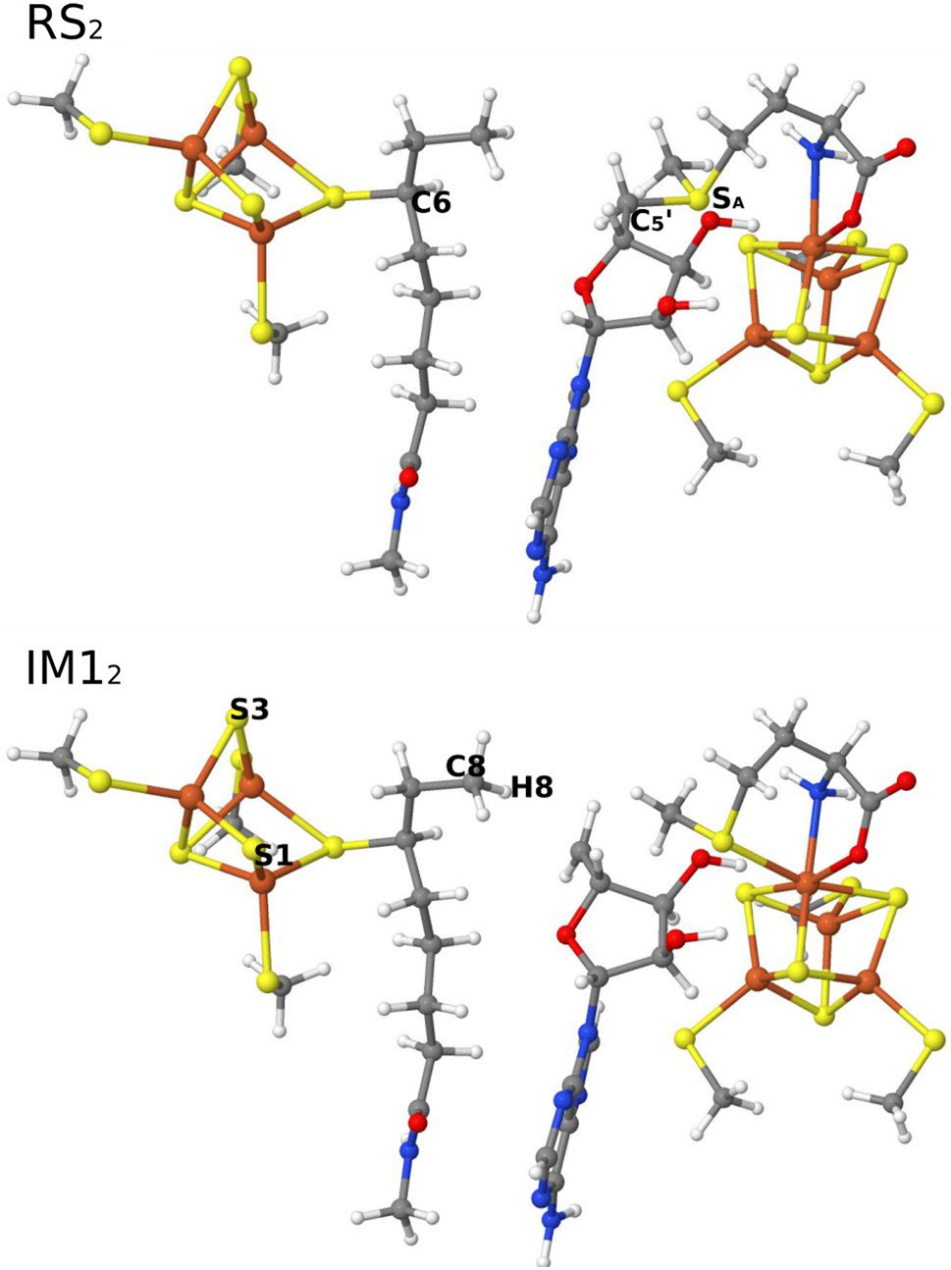
RS_2_ and IM1_2_ structures in the second S-insertion reaction

Before the insertion of the second sulfur atom to the C8 atom of the octanoyl substrate, one of the Fe^2+^ ions should dissociate. In addition, 5′-dA and Met need to be replaced by a new molecule of AdoMet and the active site should be oxidised, generating a [Fe^2+^2Fe^3+^4S^2−^]–substrate complex, which has been characterised by EPR spectroscopy [11, 12, 57]. The optimised RS_2_ species is shown in Fig. 4. The two FeS clusters are in the [2Fe^2+^2Fe^3+^4S^2−^] and [Fe^2+^2Fe^3+^4S^2−^] states. Again, the cleavage of the C_5_′–S_A_ bond of AdoMet was triggered by reducing the main FeS cluster by one electron. Interestingly, the reduction led directly to cleavage of the C_5_′–S_A_ bond without any barrier, giving rise to IM1_2_ (even if we first fixed the C_5_′–S_A_ bond length). The IM1_2_ species is also shown in Fig. 4 and has a C_5_′–H8 distance of 2.6 Å. It is 101 kJ/mol more stable than the reduced RS_2_ state (obtained by fixing the C_5_′–S_A_ bond length to 1.9 Å). We also studied the cleavage of the C_5_′–S_A_ bond of AdoMet in RS_2_ state (before the one-electron reduction): It had an energy barrier of 18 kJ/mol and the reaction was exothermic by 62 kJ/mol. Thus, one-electron reduction of RS_2_ leads to a more facile reaction mechanism, but it is not necessary from an energetic point of view.

In the next step, the C_5_′ radical abstracted the H8 atom from the octanoyl substrate. In variance to the first S-insertion reaction, we found an intermediate IM2_2_ with a radical on the C8 atom. It was 24 kJ/mol more stable than IM1_2_ and the barrier was 71 kJ/mol.

**Fig. 5:**
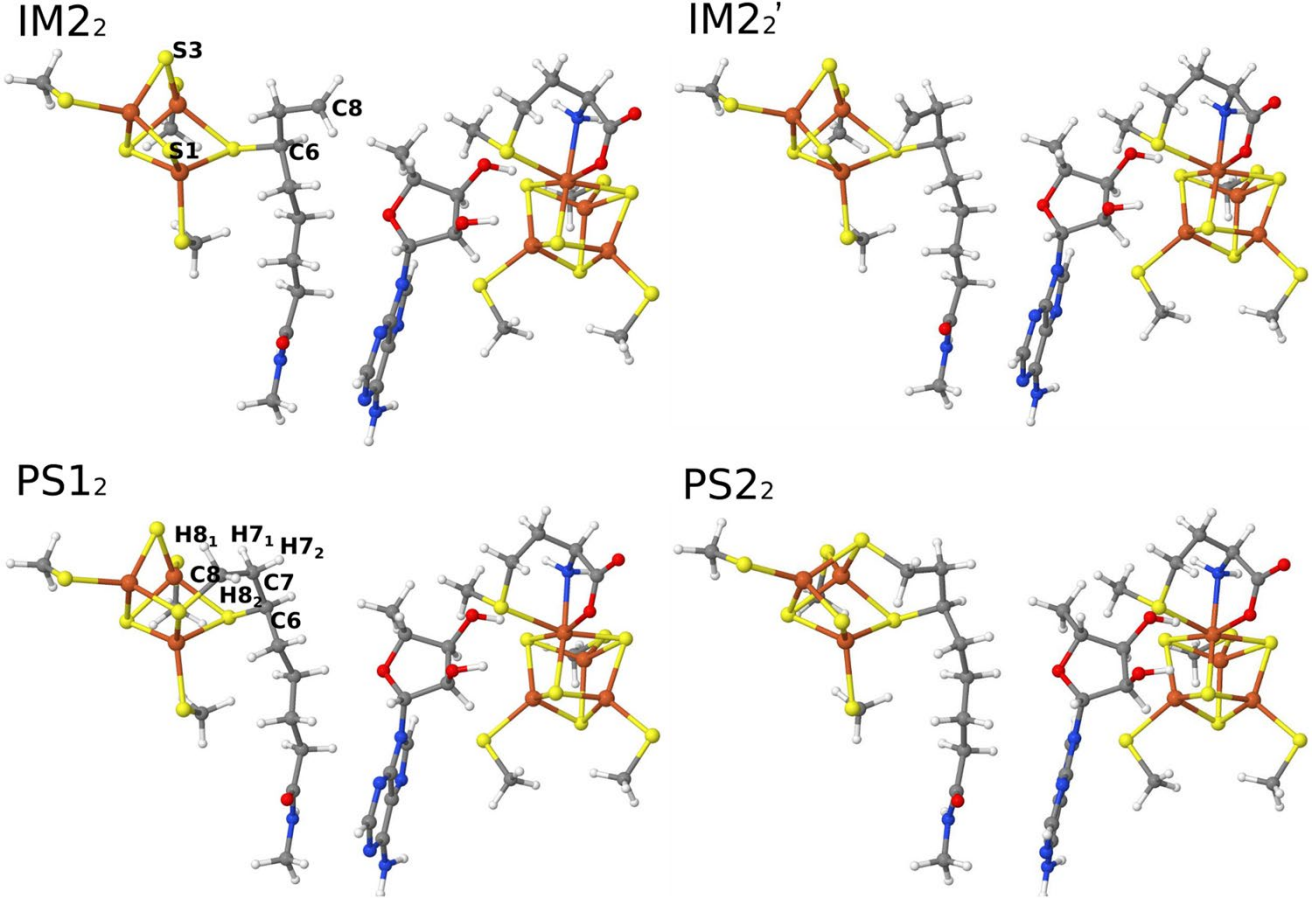
Structures of IM2_2_, IM2_2_′, PS1_2,_ and PS2_2_ in the second S-insertion reaction

**Fig. 6:**
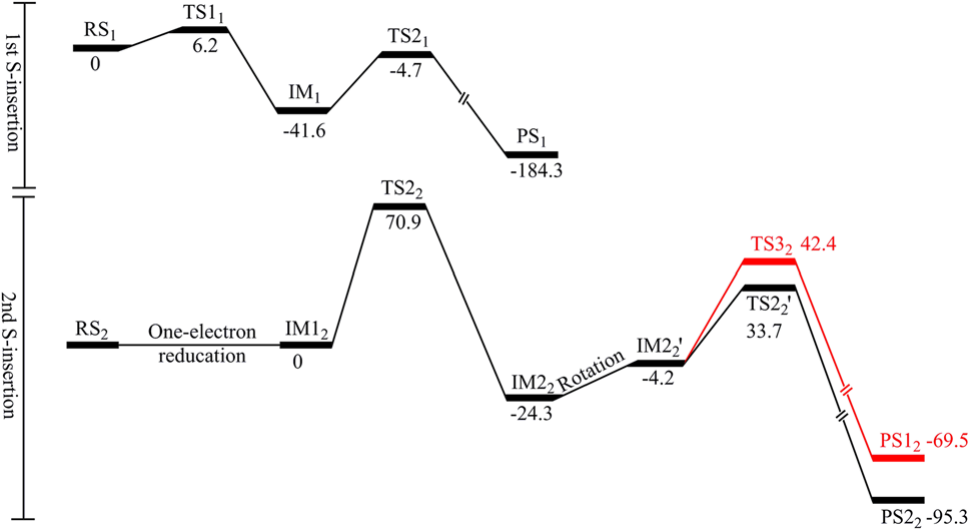
Energy profile for the two S-insertion reaction mechanisms catalysed by LipA. Energies are *E*_tot_ (Eq. 2) in kJ/mol

In the IM2_2_ state, the C8 radical can be attacked by either the S1 or S3 atom from the auxiliary FeS cluster. However, the C8 atom is quite far from both ions, 5.1 and 5.4 Å. Therefore, the substrate has to rotate around the C6–C7 bond before the reaction is possible, giving rise to a third intermediate (IM2_2_′ in Fig. 6). Since the Ser291, Ser 292, and Tyr293 residues (in the MM system) are close to the substrate, they were allowed to relax in the QM/MM calculation. The resulting IM2_2_′ state was 20 kJ/mol less stable than IM2_2_. This leads to distances of S1–C8 = 3.3 Å and S3–C8 = 3.7 Å, respectively. For the next step, two mechanisms were examined, viz. attack by S1 (mechanism 1) or S3 (mechanism 2). Our results indicate that mechanism 2 (Fig. 5) gave an 8 kJ/mol lower energy barrier (34 vs. 42 kJ/mol) and a 26 kJ/mol more stable product (− 95 vs. − 69 kJ/mol) than mechanism 1. For the two transition states (TS3_2_ and TS3_2_′), the S1–C8 distance was 2.5 Å in mechanism 1 and the S3–C8 distance of 2.8 Å in mechanism 2. In addition, the conformation of the substrate in TS3_2_ and TS3_2_′ was different: the H8_1_–C8–C7–H7_1_ and H8_2_–C8–C7–H7_2_ dihedral angles were 2° and 27° in TS3_2_, whereas they were 83° and 70° in TS3_2_′. Similar differences were also found for the two products. These structural differences probably cause the lower barrier and more stable PS2_2_ for mechanism 2.

The relative energies of all states in the reaction mechanism are collected in Fig. 6. It can be seen that the hydrogen abstraction by 5′-dA^•^ had the highest energy barrier for both reactions, 37 and 71 kJ/mol for the first and second S-insertion reactions, respectively. In addition, the two mechanisms are exothermic by 184 and 95 kJ/mol, respectively.

## Conclusion

In this paper, we have studied the reaction mechanism of lipoyl synthase with the QM/MM approach. Geometry optimisations were performed with the QM(TPSS/def2-SV(P))/MM approach. To obtain more accurate energies, single-point calculations were carried out at the B3LYP/def2-SV(P) and TPSS/def2-TZVP levels of theory.

First, we have examined the spin states of the two FeS clusters in the active site of LipA, both in the resting state and in the reactive state. The singlet [↑↑↓↓ ↓↑↑↓] spin state turned out to be the ground state. Our results indicate that the energies of the various BS states vary quite extensively. For example, if Fe5 in the auxiliary cluster of the reactive state is spin up, it will destabilise the system by 21–37 kJ/mol. In addition, the Fe–S bond distances were somewhat shorter if water was the fourth ligand or if there was no fourth ligand. On the other hand, the Fe–S bonds were slightly elongated when AdoMet bound to the Fe ion.

Second, we have investigated the mechanism for the formation of the 5′-dA^•^ radical, which is a powerful oxidant. The activation energy was 6 kJ/mol in first S-insert reaction, whereas 5′-dA^•^ was generated without any barrier when the main cluster is reduced in the second S-insertion reaction. The difference between the first and second S-insertion reactions is that one Fe ion has dissociated from the auxiliary FeS cluster in latter reaction, which changes the redox state of FeS cluster and affects the reactivity of enzyme. The substrate is also bound to one of the sulfide ions of the auxiliary cluster.

Third, our results indicated that the attack of the S^2−^ ion on the C6 radical of the octanoyl substrate takes place in the same step as the transfer of H6 from substrate to 5′-dA^•^. In the second S-insertion reaction, a C8 radical intermediate is formed and it has to change its conformation before it can react with one of the S^2−^ ions of the auxiliary cluster. Our calculations indicate that the reaction is more facile with the S3 ion than with the S1 ion and the energy barrier of the S3 ion attacking the C8 substrate radical is 30 kJ/mol. For both half-reactions, the abstraction of an H atom from the octanoyl substrate by the 5′-dA^•^ radical is rate limiting, with barriers of 37 and 71 kJ/mol, respectively.

## Electronic supplementary material

Below is the link to the electronic supplementary material.

Supplementary material 1 (PDF 164 kb)
