# Supplementary material for: Insight into the reaction mechanism of lipoyl synthase: a QM/MM study
Source: J Biol Inorg Chem. 2017 Dec 4;23(2):221–9. doi: 10.1007/s00775-017-1522-8 (PMC5816104; doi:10.1007/s00775-017-1522-8)
Supplement: Supplementary file 1 — Supplementary material 1 (PDF 164 kb) [file 775_2017_1522_MOESM1_ESM.pdf]

Electronic Supplementary Material

Insight into the Reaction Mechanism of Lipoyl Synthase: A QM/MM Study

Geng Dong*, Lili Cao, Ulf Ryde

Department of Theoretical Chemistry, Lund University, Chemical Centre, P.O. Box 124, SE-221 00, Lund, Sweden

Correspondence to Geng Dong, E-mail: Geng.Dong@teokem.lu.se,

2017-11-28

Table S1. Energy components for the various BS states of the two FeS cluster in LipA for the resting state (kJ/mol).

Fixed spin	Spin state	$E_{QM(TPSS/def2-SV(P))/MM}$	$E_{QM(TPSS/def2-SV(P))}$	$E_{QM(TPSS/def2-TZVP)}$	$E_{QM(B3LYP/def2-SV(P))}$	E_{tot}
Main cluster bound with H_2O	↑↓↑↓ ↓↑↓↑	0	0	0	0	0
	↑↓↑↓ ↑↓↓↑	3.8	5.4	9.2	-7.1	-4.7
	↑↓↑↓ ↑↑↓↓	6.2	7.4	8.7	2.6	2.7
	↑↓↑↓ ↓↓↑↑	7.1	9.0	9.3	3.5	1.9
	↑↓↑↓ ↓↑↑↓	4.9	6.1	8.1	-6.8	-5.9
	↑↓↑↓ ↑↓↑↓	0.5	0.9	-0.2	0.7	-0.9
Auxiliary FeS cluster (bound with Ser residue)	↓↑↑↓ ↓↑↑↓	2.8	0.4	-0.7	-20.6	-19.3
	↓↓↑↑ ↓↑↑↓	1.0	2.8	7.0	-21.3	-18.9
	↑↑↓↓ ↑↑↓↓	0.7	2.4	8.1	-21.8	-17.7
	↑↓↓↑ ↓↑↑↓	-0.6	-2.9	0.6	-20.2	-14.4
	↓↑↓↑ ↓↑↑↓	3.8	5.0	5.9	-7.3	-7.6

Table S2. Energy components for the various BS states of the two FeS clusters in LipA for the reactive state (kJ/mol).

Fixed spin	Spin state	$E_{QM(TPSS/def2-SV(P))/MM}$	$E_{QM(TPSS/def2-SV(P))}$	$E_{QM(TPSS/def2-TZVP)}$	$E_{QM(B3LYP/def2-SV(P))}$	E_{tot}
Main cluster bound with H_2O	↑↓↑↓ ↓↑↓↑	0	0	0	0	0
	↑↓↑↓ ↑↓↓↑	8.5	10.3	7.8	27.9	23.7
	↑↓↑↓ ↑↑↓↓	13.6	11.9	13.3	34.3	37.4
	↑↓↑↓ ↓↓↑↑	1.8	0.1	3.9	-5.1	0.4
	↑↓↑↓ ↓↑↑↓	-4.4	-3.4	-0.5	-4.1	-2.1
	↑↓↑↓ ↑↓↑↓	8.9	9.7	8.1	23.5	21.2
Auxiliary FeS cluster (bound with Ser residue)	↓↑↑↓ ↓↑↑↓	-6.3	-6.0	-1.0	-2.8	1.8
	↓↓↑↑ ↓↑↑↓	-	-	-	-	-
	↓↓↑↑ ↓↑↓↑	0.7	0.1	1.7	-0.7	1.5
	↑↑↓↓ ↓↑↑↓	-1.9	-1.9	-0.8	-10.0	-9.1
	↑↓↓↑ ↓↑↑↓	-2.1	-2.3	2.6	-4.5	0.7
	↓↑↓↑ ↓↑↑↓	-8.2	-5.8	-1.1	2.8	5.1

Table S3. Energy components for the key species in reaction mechanism (kJ/mol).

State	$E_{QM(TPSS/def2\text{-}SV(P))/MM}$	$E_{QM(TPSS/def2\text{-}SV(P))}$	$E_{QM(TPSS/def2\text{-}TZVP)}$	$E_{QM(B3LYP/def2\text{-}SV(P))}$	E_{tot}
RS_1	0	0	0	0	0
$TS1_1$	2.3	3.6	5.2	5.9	6.2
IM_1	-76.4	-49.5	-28.6	-35.6	-41.6
$TS2_1$	-42.4	-10.9	13.9	2.1	-4.7
PS_1	-185.8	-152.8	-127.7	-176.3	-184.3
$IM1_2$	0	0	0	0	0
$TS2_2$	57.5	66.8	72.8	74.1	70.9
$IM2_2$	-25.2	-24.9	-23.5	-25.5	-24.3
$IM2_2'$	-11.3	-14.1	-7.6	-13.6	-4.2
$TS3_2$	19.2	18.1	23.0	36.4	42.4
$PS1_2$	-64.7	-72.0	-67.5	-81.3	-69.5
$TS3_2'$	20.3	1.7	6.4	10.3	33.7
$PS2_2$	-78.3	-105.0	-101.3	-125.7	-95.3

Cartesian coordinates of optimised structures:

RS_1 (QM region of QM/MM calculation)

ATOM	1	H	???	1	-4.420	8.948	-3.812
ATOM	2	C	???	1	-5.271	9.291	-4.462
ATOM	3	H	???	1	-4.904	9.404	-5.499
ATOM	4	H	???	1	-5.614	10.278	-4.100
ATOM	5	S	???	1	-6.740	8.172	-4.441
ATOM	6	H	???	1	-0.244	7.480	-6.146
ATOM	7	C	???	1	-1.247	7.018	-6.038
ATOM	8	H	???	1	-2.022	7.800	-6.142
ATOM	9	H	???	1	-1.325	6.572	-5.030
ATOM	10	S	???	1	-1.446	5.703	-7.336
ATOM	11	H	???	1	-8.127	7.587	-11.043
ATOM	12	C	???	1	-7.750	7.163	-10.090
ATOM	13	H	???	1	-8.581	7.025	-9.378
ATOM	14	H	???	1	-7.002	7.852	-9.654
ATOM	15	S	???	1	-6.972	5.519	-10.439
ATOM	16	H	???	1	0.565	6.360	10.259
ATOM	17	C	???	1	0.874	5.763	9.362
ATOM	18	H	???	1	1.941	5.961	9.160
ATOM	19	H	???	1	0.273	6.122	8.506
ATOM	20	S	???	1	0.601	3.945	9.608
ATOM	21	H	???	1	6.567	0.591	5.844
ATOM	22	C	???	1	5.645	0.163	5.392
ATOM	23	H	???	1	5.919	-0.670	4.716
ATOM	24	H	???	1	5.142	0.945	4.795
ATOM	25	S	???	1	4.491	-0.447	6.710
ATOM	26	H	???	1	5.101	3.983	2.374
ATOM	27	C	???	1	4.425	3.551	3.143
ATOM	28	H	???	1	4.915	3.589	4.132
ATOM	29	H	???	1	4.230	2.495	2.879
ATOM	30	S	???	1	2.820	4.485	3.185
ATOM	31	H	???	1	5.340	6.388	-4.982
ATOM	32	C	???	1	4.551	5.783	-5.450
ATOM	33	H	???	1	4.443	6.089	-6.512
ATOM	34	H	???	1	4.875	4.727	-5.420
ATOM	35	N	???	1	3.310	5.897	-4.713
ATOM	36	H	???	1	3.129	6.679	-4.076
ATOM	37	C	???	1	-2.662	2.897	3.457
ATOM	38	H	???	1	-2.473	2.898	4.544
ATOM	39	H	???	1	-2.197	3.799	3.027
ATOM	40	H	???	1	-3.734	2.814	3.212
ATOM	41	S	???	1	-1.755	1.462	2.785
ATOM	42	C	???	1	-2.906	0.056	3.227
ATOM	43	H	???	1	-2.321	-0.826	2.945
ATOM	44	H	???	1	-3.747	0.172	2.526
ATOM	45	C	???	1	-3.484	-0.066	4.653

```
ATOM     46  H   ???     1      -4.150  -0.946   4.553
ATOM     47  H   ???     1      -4.140   0.795   4.871
ATOM     48  C   ???     1      -2.616  -0.305   5.907
ATOM     49  N   ???     1      -2.046   0.974   6.375
ATOM     50  H   ???     1      -2.743   1.739   6.348
ATOM     51  H   ???     1      -1.750   0.895   7.361
ATOM     52  H   ???     1      -3.308  -0.731   6.659
ATOM     53  C   ???     1      -1.472  -1.338   5.707
ATOM     54  O   ???     1      -0.367  -0.857   5.262
ATOM     55  O   ???     1      -1.691  -2.540   5.961
ATOM     56 FE   ???     1      -5.761   4.047  -6.043
ATOM     57  S   ???     1      -4.662   3.545  -7.979
ATOM     58 FE   ???     1      -3.683   5.415  -6.918
ATOM     59  S   ???     1      -4.369   5.266  -4.721
ATOM     60 FE   ???     1      -6.199   5.310  -8.327
ATOM     61  S   ???     1      -4.958   7.228  -7.684
ATOM     62 FE   ???     1      -6.007   6.527  -5.838
ATOM     63  S   ???     1      -7.709   5.068  -6.585
ATOM     64 FE   ???     1       0.140   1.207   5.756
ATOM     65  S   ???     1      -0.157   3.618   5.836
ATOM     66 FE   ???     1       1.222   3.066   7.591
ATOM     67  S   ???     1       2.003   1.010   4.298
ATOM     68 FE   ???     1       2.839   1.111   6.437
ATOM     69  S   ???     1       3.379   3.360   6.737
ATOM     70 FE   ???     1       1.969   3.254   4.964
ATOM     71  S   ???     1       0.983   0.738   7.843
ATOM     72  C   ???     1       2.629   1.346  -2.900
ATOM     73  N   ???     1       2.749   0.472  -3.922
ATOM     74  C   ???     1       3.862   0.678  -4.648
ATOM     75  H   ???     1       3.985   0.019  -5.521
ATOM     76  N   ???     1       4.863   1.566  -4.462
ATOM     77  C   ???     1       4.757   2.403  -3.400
ATOM     78  N   ???     1       5.730   3.302  -3.114
ATOM     79  H   ???     1       5.777   3.678  -2.158
ATOM     80  H   ???     1       6.582   3.328  -3.687
ATOM     81  C   ???     1       3.581   2.344  -2.611
ATOM     82  N   ???     1       3.167   3.165  -1.585
ATOM     83  C   ???     1       1.999   2.669  -1.233
ATOM     84  H   ???     1       1.325   3.106  -0.512
ATOM     85  N   ???     1       1.608   1.556  -1.958
ATOM     86  C   ???     1       0.361   0.814  -1.668
ATOM     87  O   ???     1      -0.507   1.727  -0.976
ATOM     88  H   ???     1      -0.074   0.497  -2.634
ATOM     89  C   ???     1       0.525  -0.434  -0.745
ATOM     90  O   ???     1       0.173  -1.600  -1.452
ATOM     91  H   ???     1       0.370  -2.352  -0.804
ATOM     92  H   ???     1       1.560  -0.484  -0.359
ATOM     93  C   ???     1      -0.477  -0.151   0.425
ATOM     94  O   ???     1      -1.368  -1.208   0.742
ATOM     95  H   ???     1      -1.009  -1.667   1.562
```

ATOM	96	H	???	1	0.081	0.199	1.316
ATOM	97	C	???	1	-1.363	0.950	-0.124
ATOM	98	H	???	1	-2.134	0.453	-0.747
ATOM	99	C	???	1	-2.101	1.803	0.932
ATOM	100	H	???	1	-3.197	1.736	0.829
ATOM	101	H	???	1	-1.782	2.850	0.831
ATOM	102	O	???	1	2.599	3.992	-5.676
ATOM	103	C	???	1	2.394	4.907	-4.859
ATOM	104	C	???	1	1.181	4.962	-3.954
ATOM	105	H	???	1	0.788	5.995	-3.910
ATOM	106	H	???	1	1.542	4.727	-2.932
ATOM	107	C	???	1	0.101	3.974	-4.378
ATOM	108	H	???	1	0.550	2.961	-4.383
ATOM	109	H	???	1	-0.229	4.203	-5.413
ATOM	110	C	???	1	-1.109	3.981	-3.446
ATOM	111	H	???	1	-1.620	4.964	-3.483
ATOM	112	H	???	1	-0.776	3.821	-2.400
ATOM	113	C	???	1	-2.113	2.892	-3.828
ATOM	114	H	???	1	-1.583	1.917	-3.906
ATOM	115	H	???	1	-2.538	3.127	-4.826
ATOM	116	C	???	1	-3.239	2.758	-2.808
ATOM	117	H	???	1	-3.750	3.733	-2.698
ATOM	118	H	???	1	-2.786	2.517	-1.824
ATOM	119	C	???	1	-4.273	1.686	-3.165
ATOM	120	H	???	1	-3.737	0.798	-3.572
ATOM	121	H	???	1	-4.927	2.075	-3.978
ATOM	122	C	???	1	-5.102	1.293	-1.942
ATOM	123	H	???	1	-5.769	0.433	-2.121
ATOM	124	H	???	1	-4.410	1.032	-1.116
ATOM	125	H	???	1	-5.735	2.135	-1.611

END

IM_1 (QM region of QM/MM calculation)

ATOM	1	H	???	1	-4.419	8.952	-3.814
ATOM	2	C	???	1	-5.264	9.304	-4.467
ATOM	3	H	???	1	-4.888	9.439	-5.498
ATOM	4	H	???	1	-5.613	10.283	-4.090
ATOM	5	S	???	1	-6.740	8.199	-4.488
ATOM	6	H	???	1	-0.249	7.483	-6.142
ATOM	7	C	???	1	-1.259	7.032	-6.026
ATOM	8	H	???	1	-2.019	7.832	-6.095
ATOM	9	H	???	1	-1.318	6.562	-5.029
ATOM	10	S	???	1	-1.529	5.751	-7.342
ATOM	11	H	???	1	-8.126	7.590	-11.038
ATOM	12	C	???	1	-7.750	7.175	-10.077
ATOM	13	H	???	1	-8.584	7.053	-9.366
ATOM	14	H	???	1	-7.001	7.871	-9.652
ATOM	15	S	???	1	-6.970	5.524	-10.368

ATOM	16	H	???	1	0.564	6.362	10.259
ATOM	17	C	???	1	0.872	5.765	9.363
ATOM	18	H	???	1	1.938	5.966	9.156
ATOM	19	H	???	1	0.265	6.114	8.508
ATOM	20	S	???	1	0.606	3.952	9.631
ATOM	21	H	???	1	6.564	0.593	5.844
ATOM	22	C	???	1	5.638	0.171	5.392
ATOM	23	H	???	1	5.904	-0.657	4.708
ATOM	24	H	???	1	5.131	0.959	4.807
ATOM	25	S	???	1	4.491	-0.446	6.710
ATOM	26	H	???	1	5.105	3.980	2.379
ATOM	27	C	???	1	4.437	3.541	3.155
ATOM	28	H	???	1	4.950	3.564	4.134
ATOM	29	H	???	1	4.238	2.488	2.880
ATOM	30	S	???	1	2.834	4.467	3.247
ATOM	31	H	???	1	5.336	6.387	-4.980
ATOM	32	C	???	1	4.542	5.782	-5.442
ATOM	33	H	???	1	4.415	6.100	-6.499
ATOM	34	H	???	1	4.871	4.728	-5.430
ATOM	35	N	???	1	3.310	5.885	-4.686
ATOM	36	H	???	1	3.146	6.646	-4.019
ATOM	37	C	???	1	-2.561	2.785	3.490
ATOM	38	H	???	1	-2.998	2.935	4.490
ATOM	39	H	???	1	-1.848	3.603	3.289
ATOM	40	H	???	1	-3.356	2.761	2.722
ATOM	41	S	???	1	-1.579	1.250	3.406
ATOM	42	C	???	1	-2.878	-0.038	3.590
ATOM	43	H	???	1	-2.363	-0.960	3.283
ATOM	44	H	???	1	-3.643	0.180	2.824
ATOM	45	C	???	1	-3.543	-0.233	4.957
ATOM	46	H	???	1	-4.160	-1.150	4.875
ATOM	47	H	???	1	-4.236	0.601	5.174
ATOM	48	C	???	1	-2.611	-0.404	6.177
ATOM	49	N	???	1	-2.053	0.906	6.595
ATOM	50	H	???	1	-2.735	1.669	6.449
ATOM	51	H	???	1	-1.817	0.897	7.597
ATOM	52	H	???	1	-3.234	-0.830	6.980
ATOM	53	C	???	1	-1.475	-1.425	5.892
ATOM	54	O	???	1	-0.360	-0.947	5.462
ATOM	55	O	???	1	-1.713	-2.641	6.046
ATOM	56	FE	???	1	-5.853	4.083	-5.980
ATOM	57	S	???	1	-4.752	3.546	-7.888
ATOM	58	FE	???	1	-3.738	5.436	-6.906
ATOM	59	S	???	1	-4.456	5.387	-4.748
ATOM	60	FE	???	1	-6.248	5.296	-8.253
ATOM	61	S	???	1	-5.023	7.220	-7.749
ATOM	62	FE	???	1	-6.088	6.579	-5.923
ATOM	63	S	???	1	-7.792	5.113	-6.578
ATOM	64	FE	???	1	1.983	3.234	5.017
ATOM	65	S	???	1	-0.120	3.644	5.898

ATOM	66	FE	???	1	2.775	1.075	6.459
ATOM	67	S	???	1	2.003	1.010	4.343
ATOM	68	FE	???	1	1.219	3.042	7.619
ATOM	69	S	???	1	3.393	3.299	6.793
ATOM	70	FE	???	1	0.009	1.189	5.713
ATOM	71	S	???	1	0.974	0.731	7.890
ATOM	72	C	???	1	2.783	1.308	-3.045
ATOM	73	N	???	1	2.950	0.487	-4.106
ATOM	74	C	???	1	4.056	0.793	-4.804
ATOM	75	H	???	1	4.227	0.185	-5.706
ATOM	76	N	???	1	5.000	1.729	-4.554
ATOM	77	C	???	1	4.837	2.506	-3.450
ATOM	78	N	???	1	5.762	3.434	-3.101
ATOM	79	H	???	1	5.793	3.730	-2.116
ATOM	80	H	???	1	6.633	3.476	-3.643
ATOM	81	C	???	1	3.660	2.348	-2.683
ATOM	82	N	???	1	3.183	3.064	-1.600
ATOM	83	C	???	1	2.051	2.451	-1.295
ATOM	84	H	???	1	1.353	2.747	-0.518
ATOM	85	N	???	1	1.756	1.378	-2.113
ATOM	86	C	???	1	0.536	0.562	-2.008
ATOM	87	O	???	1	-0.526	1.454	-1.727
ATOM	88	H	???	1	0.426	0.067	-2.990
ATOM	89	C	???	1	0.554	-0.517	-0.893
ATOM	90	O	???	1	0.181	-1.745	-1.482
ATOM	91	H	???	1	0.346	-2.431	-0.751
ATOM	92	H	???	1	1.550	-0.576	-0.414
ATOM	93	C	???	1	-0.529	-0.027	0.122
ATOM	94	O	???	1	-1.341	-1.059	0.648
ATOM	95	H	???	1	-0.964	-1.378	1.513
ATOM	96	H	???	1	-0.076	0.588	0.922
ATOM	97	C	???	1	-1.431	0.858	-0.766
ATOM	98	H	???	1	-2.127	0.167	-1.292
ATOM	99	C	???	1	-2.164	1.912	-0.030
ATOM	100	H	???	1	-3.086	1.646	0.497
ATOM	101	H	???	1	-1.626	2.822	0.254
ATOM	102	O	???	1	2.562	4.027	-5.711
ATOM	103	C	???	1	2.379	4.910	-4.855
ATOM	104	C	???	1	1.171	4.947	-3.940
ATOM	105	H	???	1	0.799	5.987	-3.855
ATOM	106	H	???	1	1.525	4.664	-2.928
ATOM	107	C	???	1	0.072	3.996	-4.404
ATOM	108	H	???	1	0.485	2.968	-4.385
ATOM	109	H	???	1	-0.201	4.226	-5.454
ATOM	110	C	???	1	-1.182	4.035	-3.529
ATOM	111	H	???	1	-1.655	5.038	-3.570
ATOM	112	H	???	1	-0.902	3.845	-2.475
ATOM	113	C	???	1	-2.193	2.975	-3.973
ATOM	114	H	???	1	-1.664	2.003	-4.044
ATOM	115	H	???	1	-2.581	3.234	-4.982

```
ATOM   116  C   ???   1      -3.344   2.814  -2.985
ATOM   117  H   ???   1      -3.905   3.762  -2.893
ATOM   118  H   ???   1      -2.911   2.615  -1.982
ATOM   119  C   ???   1      -4.313   1.676  -3.325
ATOM   120  H   ???   1      -3.724   0.818  -3.722
ATOM   121  H   ???   1      -4.993   2.006  -4.150
ATOM   122  C   ???   1      -5.109   1.241  -2.092
ATOM   123  H   ???   1      -5.752   0.363  -2.272
ATOM   124  H   ???   1      -4.400   0.988  -1.283
ATOM   125  H   ???   1      -5.761   2.058  -1.735
END
```

PS_1 (QM region of QM/MM calculation)

```
ATOM     1  H   ???   1      -4.422   8.949  -3.808
ATOM     2  C   ???   1      -5.275   9.293  -4.451
ATOM     3  H   ???   1      -4.925   9.394  -5.494
ATOM     4  H   ???   1      -5.615  10.281  -4.090
ATOM     5  S   ???   1      -6.743   8.171  -4.386
ATOM     6  H   ???   1      -0.246   7.482  -6.151
ATOM     7  C   ???   1      -1.254   7.026  -6.051
ATOM     8  H   ???   1      -2.025   7.810  -6.169
ATOM     9  H   ???   1      -1.340   6.590  -5.042
ATOM    10  S   ???   1      -1.459   5.698  -7.335
ATOM    11  H   ???   1      -8.124   7.589 -11.039
ATOM    12  C   ???   1      -7.744   7.170 -10.082
ATOM    13  H   ???   1      -8.579   7.009  -9.379
ATOM    14  H   ???   1      -7.018   7.876  -9.637
ATOM    15  S   ???   1      -6.917   5.547 -10.421
ATOM    16  H   ???   1       0.565   6.361  10.259
ATOM    17  C   ???   1       0.873   5.763   9.363
ATOM    18  H   ???   1       1.939   5.968   9.155
ATOM    19  H   ???   1       0.266   6.112   8.508
ATOM    20  S   ???   1       0.613   3.948   9.626
ATOM    21  H   ???   1       6.564   0.593   5.845
ATOM    22  C   ???   1       5.637   0.169   5.395
ATOM    23  H   ???   1       5.903  -0.662   4.714
ATOM    24  H   ???   1       5.132   0.955   4.805
ATOM    25  S   ???   1       4.489  -0.442   6.715
ATOM    26  H   ???   1       5.104   3.980   2.378
ATOM    27  C   ???   1       4.435   3.543   3.154
ATOM    28  H   ???   1       4.943   3.570   4.135
ATOM    29  H   ???   1       4.236   2.490   2.882
ATOM    30  S   ???   1       2.833   4.473   3.237
ATOM    31  H   ???   1       5.333   6.386  -4.979
ATOM    32  C   ???   1       4.536   5.779  -5.437
ATOM    33  H   ???   1       4.394   6.107  -6.488
ATOM    34  H   ???   1       4.871   4.727  -5.440
ATOM    35  N   ???   1       3.311   5.862  -4.665
ATOM    36  H   ???   1       3.138   6.631  -4.008
```

ATOM	37	C	???	1	-2.550	2.765	3.475
ATOM	38	H	???	1	-3.046	2.899	4.450
ATOM	39	H	???	1	-1.821	3.582	3.335
ATOM	40	H	???	1	-3.299	2.762	2.662
ATOM	41	S	???	1	-1.572	1.226	3.421
ATOM	42	C	???	1	-2.881	-0.053	3.600
ATOM	43	H	???	1	-2.373	-0.980	3.297
ATOM	44	H	???	1	-3.643	0.168	2.832
ATOM	45	C	???	1	-3.549	-0.239	4.967
ATOM	46	H	???	1	-4.169	-1.154	4.888
ATOM	47	H	???	1	-4.238	0.598	5.181
ATOM	48	C	???	1	-2.617	-0.409	6.187
ATOM	49	N	???	1	-2.060	0.900	6.608
ATOM	50	H	???	1	-2.737	1.666	6.451
ATOM	51	H	???	1	-1.838	0.894	7.613
ATOM	52	H	???	1	-3.240	-0.837	6.989
ATOM	53	C	???	1	-1.479	-1.429	5.901
ATOM	54	O	???	1	-0.363	-0.948	5.479
ATOM	55	O	???	1	-1.717	-2.646	6.047
ATOM	56	FE	???	1	-5.756	4.095	-6.016
ATOM	57	S	???	1	-4.665	3.514	-7.913
ATOM	58	FE	???	1	-3.681	5.376	-6.938
ATOM	59	S	???	1	-4.199	5.263	-4.595
ATOM	60	FE	???	1	-6.156	5.314	-8.321
ATOM	61	S	???	1	-4.906	7.227	-7.566
ATOM	62	FE	???	1	-6.068	6.539	-5.794
ATOM	63	S	???	1	-7.698	5.048	-6.612
ATOM	64	FE	???	1	1.973	3.247	5.006
ATOM	65	S	???	1	-0.126	3.644	5.894
ATOM	66	FE	???	1	2.785	1.097	6.455
ATOM	67	S	???	1	1.990	1.020	4.342
ATOM	68	FE	???	1	1.233	3.047	7.607
ATOM	69	S	???	1	3.395	3.326	6.768
ATOM	70	FE	???	1	0.003	1.176	5.748
ATOM	71	S	???	1	0.982	0.741	7.892
ATOM	72	C	???	1	2.796	1.305	-3.062
ATOM	73	N	???	1	2.968	0.491	-4.126
ATOM	74	C	???	1	4.075	0.805	-4.819
ATOM	75	H	???	1	4.251	0.204	-5.724
ATOM	76	N	???	1	5.013	1.744	-4.558
ATOM	77	C	???	1	4.845	2.514	-3.449
ATOM	78	N	???	1	5.766	3.442	-3.095
ATOM	79	H	???	1	5.796	3.734	-2.108
ATOM	80	H	???	1	6.637	3.489	-3.636
ATOM	81	C	???	1	3.666	2.345	-2.686
ATOM	82	N	???	1	3.181	3.048	-1.597
ATOM	83	C	???	1	2.050	2.426	-1.303
ATOM	84	H	???	1	1.352	2.707	-0.520
ATOM	85	N	???	1	1.764	1.362	-2.136
ATOM	86	C	???	1	0.556	0.532	-2.062

ATOM	87	O	???	1	-0.544	1.409	-1.875
ATOM	88	H	???	1	0.509	-0.004	-3.027
ATOM	89	C	???	1	0.531	-0.504	-0.908
ATOM	90	O	???	1	0.165	-1.749	-1.471
ATOM	91	H	???	1	0.344	-2.425	-0.732
ATOM	92	H	???	1	1.513	-0.559	-0.400
ATOM	93	C	???	1	-0.584	0.029	0.046
ATOM	94	O	???	1	-1.380	-0.992	0.624
ATOM	95	H	???	1	-0.975	-1.291	1.481
ATOM	96	H	???	1	-0.156	0.695	0.821
ATOM	97	C	???	1	-1.464	0.842	-0.910
ATOM	98	H	???	1	-2.136	0.128	-1.432
ATOM	99	C	???	1	-2.256	1.961	-0.262
ATOM	100	H	???	1	-3.006	1.523	0.422
ATOM	101	H	???	1	-1.584	2.611	0.325
ATOM	102	O	???	1	2.572	3.993	-5.679
ATOM	103	C	???	1	2.389	4.877	-4.825
ATOM	104	C	???	1	1.182	4.911	-3.907
ATOM	105	H	???	1	0.808	5.951	-3.834
ATOM	106	H	???	1	1.536	4.637	-2.893
ATOM	107	C	???	1	0.072	3.966	-4.368
ATOM	108	H	???	1	0.419	2.921	-4.257
ATOM	109	H	???	1	-0.132	4.147	-5.443
ATOM	110	C	???	1	-1.227	4.164	-3.587
ATOM	111	H	???	1	-1.551	5.221	-3.675
ATOM	112	H	???	1	-1.061	3.961	-2.511
ATOM	113	C	???	1	-2.351	3.258	-4.103
ATOM	114	H	???	1	-2.148	2.222	-3.766
ATOM	115	H	???	1	-2.369	3.268	-5.213
ATOM	116	C	???	1	-3.732	3.711	-3.619
ATOM	117	H	???	1	-3.662	4.045	-2.568
ATOM	118	H	???	1	-2.775	2.571	-1.021
ATOM	119	C	???	1	-4.845	2.666	-3.752
ATOM	120	H	???	1	-4.852	2.319	-4.820
ATOM	121	H	???	1	-5.824	3.142	-3.532
ATOM	122	C	???	1	-4.666	1.464	-2.811
ATOM	123	H	???	1	-5.466	0.717	-2.937
ATOM	124	H	???	1	-3.681	0.980	-2.951
ATOM	125	H	???	1	-4.732	1.797	-1.760

END

IM1$_2$ (QM region of QM/MM calculation)

ATOM	1	H	???	1	-4.423	8.939	-3.812
ATOM	2	C	???	1	-5.280	9.263	-4.463
ATOM	3	H	???	1	-4.905	9.399	-5.495
ATOM	4	H	???	1	-5.656	10.232	-4.085
ATOM	5	S	???	1	-6.713	8.097	-4.495
ATOM	6	H	???	1	-0.244	7.484	-6.157

ATOM	7	C	???	1	-1.254	7.031	-6.068
ATOM	8	H	???	1	-2.023	7.821	-6.167
ATOM	9	H	???	1	-1.348	6.579	-5.066
ATOM	10	S	???	1	-1.472	5.738	-7.379
ATOM	11	H	???	1	-8.120	7.586	-11.027
ATOM	12	C	???	1	-7.735	7.169	-10.056
ATOM	13	H	???	1	-8.573	7.008	-9.356
ATOM	14	H	???	1	-7.037	7.902	-9.607
ATOM	15	S	???	1	-6.851	5.568	-10.319
ATOM	16	H	???	1	0.564	6.358	10.261
ATOM	17	C	???	1	0.871	5.755	9.368
ATOM	18	H	???	1	1.936	5.959	9.155
ATOM	19	H	???	1	0.263	6.097	8.511
ATOM	20	S	???	1	0.611	3.941	9.648
ATOM	21	H	???	1	6.564	0.592	5.847
ATOM	22	C	???	1	5.637	0.165	5.402
ATOM	23	H	???	1	5.903	-0.660	4.713
ATOM	24	H	???	1	5.120	0.951	4.823
ATOM	25	S	???	1	4.505	-0.459	6.731
ATOM	26	H	???	1	5.104	3.980	2.379
ATOM	27	C	???	1	4.435	3.542	3.156
ATOM	28	H	???	1	4.947	3.564	4.135
ATOM	29	H	???	1	4.235	2.490	2.881
ATOM	30	S	???	1	2.833	4.471	3.248
ATOM	31	H	???	1	5.338	6.388	-4.982
ATOM	32	C	???	1	4.546	5.784	-5.448
ATOM	33	H	???	1	4.418	6.109	-6.502
ATOM	34	H	???	1	4.878	4.731	-5.444
ATOM	35	N	???	1	3.319	5.871	-4.687
ATOM	36	H	???	1	3.125	6.657	-4.060
ATOM	37	C	???	1	-2.524	2.760	3.436
ATOM	38	H	???	1	-3.077	2.866	4.383
ATOM	39	H	???	1	-1.785	3.576	3.365
ATOM	40	H	???	1	-3.222	2.784	2.578
ATOM	41	S	???	1	-1.550	1.218	3.396
ATOM	42	C	???	1	-2.869	-0.052	3.580
ATOM	43	H	???	1	-2.371	-0.984	3.277
ATOM	44	H	???	1	-3.630	0.176	2.813
ATOM	45	C	???	1	-3.536	-0.233	4.948
ATOM	46	H	???	1	-4.162	-1.144	4.871
ATOM	47	H	???	1	-4.221	0.609	5.162
ATOM	48	C	???	1	-2.607	-0.405	6.171
ATOM	49	N	???	1	-2.043	0.902	6.584
ATOM	50	H	???	1	-2.718	1.670	6.433
ATOM	51	H	???	1	-1.802	0.897	7.585
ATOM	52	H	???	1	-3.234	-0.827	6.975
ATOM	53	C	???	1	-1.474	-1.433	5.892
ATOM	54	O	???	1	-0.360	-0.961	5.464
ATOM	55	O	???	1	-1.718	-2.650	6.054
ATOM	56	S	???	1	-4.427	3.585	-8.187

```
ATOM     57 FE   ???    1      -3.727   5.330  -6.956
ATOM     58  S   ???    1      -4.028   5.467  -4.622
ATOM     59 FE   ???    1      -5.990   5.283  -8.185
ATOM     60  S   ???    1      -4.866   7.254  -7.480
ATOM     61 FE   ???    1      -5.947   6.335  -5.755
ATOM     62  S   ???    1      -7.483   5.025  -6.543
ATOM     63 FE   ???    1       2.782   1.074   6.488
ATOM     64  S   ???    1      -0.119   3.639   5.911
ATOM     65 FE   ???    1       1.982   3.225   5.020
ATOM     66  S   ???    1       2.016   0.993   4.359
ATOM     67 FE   ???    1       0.021   1.190   5.720
ATOM     68  S   ???    1       3.404   3.306   6.793
ATOM     69 FE   ???    1       1.235   3.023   7.626
ATOM     70  S   ???    1       0.965   0.715   7.905
ATOM     71  C   ???    1       2.791   1.301  -3.070
ATOM     72  N   ???    1       2.973   0.483  -4.130
ATOM     73  C   ???    1       4.083   0.794  -4.816
ATOM     74  H   ???    1       4.265   0.189  -5.718
ATOM     75  N   ???    1       5.020   1.734  -4.552
ATOM     76  C   ???    1       4.841   2.507  -3.448
ATOM     77  N   ???    1       5.757   3.444  -3.095
ATOM     78  H   ???    1       5.782   3.739  -2.110
ATOM     79  H   ???    1       6.629   3.492  -3.635
ATOM     80  C   ???    1       3.661   2.338  -2.688
ATOM     81  N   ???    1       3.170   3.041  -1.603
ATOM     82  C   ???    1       2.033   2.423  -1.319
ATOM     83  H   ???    1       1.329   2.707  -0.541
ATOM     84  N   ???    1       1.752   1.360  -2.155
ATOM     85  C   ???    1       0.547   0.522  -2.092
ATOM     86  O   ???    1      -0.563   1.386  -1.929
ATOM     87  H   ???    1       0.521  -0.021  -3.053
ATOM     88  C   ???    1       0.523  -0.502  -0.928
ATOM     89  O   ???    1       0.174  -1.756  -1.478
ATOM     90  H   ???    1       0.346  -2.421  -0.728
ATOM     91  H   ???    1       1.500  -0.541  -0.407
ATOM     92  C   ???    1      -0.604   0.029   0.012
ATOM     93  O   ???    1      -1.379  -0.995   0.605
ATOM     94  H   ???    1      -0.977  -1.261   1.473
ATOM     95  H   ???    1      -0.198   0.724   0.772
ATOM     96  C   ???    1      -1.501   0.814  -0.983
ATOM     97  H   ???    1      -2.142   0.065  -1.498
ATOM     98  C   ???    1      -2.310   1.891  -0.371
ATOM     99  H   ???    1      -3.248   1.634   0.134
ATOM    100  H   ???    1      -1.818   2.846  -0.152
ATOM    101  O   ???    1       2.626   3.959  -5.648
ATOM    102  C   ???    1       2.413   4.866  -4.826
ATOM    103  C   ???    1       1.200   4.914  -3.925
ATOM    104  H   ???    1       0.842   5.959  -3.848
ATOM    105  H   ???    1       1.537   4.626  -2.909
ATOM    106  C   ???    1       0.082   3.997  -4.413
```

ATOM	107	H	???	1	0.417	2.944	-4.329
ATOM	108	H	???	1	-0.118	4.212	-5.483
ATOM	109	C	???	1	-1.217	4.191	-3.635
ATOM	110	H	???	1	-1.515	5.257	-3.679
ATOM	111	H	???	1	-1.072	3.933	-2.567
ATOM	112	C	???	1	-2.344	3.336	-4.220
ATOM	113	H	???	1	-2.186	2.291	-3.891
ATOM	114	H	???	1	-2.307	3.364	-5.328
ATOM	115	C	???	1	-3.737	3.819	-3.799
ATOM	116	H	???	1	-3.710	4.062	-2.719
ATOM	117	C	???	1	-4.848	2.797	-4.036
ATOM	118	H	???	1	-4.939	2.616	-5.128
ATOM	119	H	???	1	-5.807	3.233	-3.702
ATOM	120	C	???	1	-4.552	1.502	-3.250
ATOM	121	H	???	1	-5.454	0.889	-3.085
ATOM	122	H	???	1	-3.771	0.885	-3.742
ATOM	123	H	???	1	-4.161	1.746	-2.241
END							

IM2$_2$ (QM region of QM/MM calculation)

ATOM	1	H	???	1	-4.423	8.939	-3.811
ATOM	2	C	???	1	-5.279	9.265	-4.462
ATOM	3	H	???	1	-4.905	9.402	-5.494
ATOM	4	H	???	1	-5.656	10.233	-4.083
ATOM	5	S	???	1	-6.710	8.097	-4.496
ATOM	6	H	???	1	-0.243	7.484	-6.157
ATOM	7	C	???	1	-1.251	7.030	-6.069
ATOM	8	H	???	1	-2.021	7.819	-6.164
ATOM	9	H	???	1	-1.343	6.574	-5.068
ATOM	10	S	???	1	-1.465	5.740	-7.384
ATOM	11	H	???	1	-8.120	7.587	-11.028
ATOM	12	C	???	1	-7.735	7.170	-10.056
ATOM	13	H	???	1	-8.573	7.011	-9.356
ATOM	14	H	???	1	-7.035	7.903	-9.608
ATOM	15	S	???	1	-6.854	5.567	-10.317
ATOM	16	H	???	1	0.564	6.358	10.261
ATOM	17	C	???	1	0.871	5.755	9.368
ATOM	18	H	???	1	1.936	5.959	9.156
ATOM	19	H	???	1	0.263	6.097	8.511
ATOM	20	S	???	1	0.610	3.942	9.649
ATOM	21	H	???	1	6.564	0.592	5.847
ATOM	22	C	???	1	5.637	0.165	5.403
ATOM	23	H	???	1	5.902	-0.660	4.714
ATOM	24	H	???	1	5.120	0.951	4.824
ATOM	25	S	???	1	4.506	-0.459	6.732
ATOM	26	H	???	1	5.104	3.980	2.379
ATOM	27	C	???	1	4.436	3.542	3.156
ATOM	28	H	???	1	4.949	3.563	4.135

ATOM	29	H	???	1	4.235	2.490	2.881
ATOM	30	S	???	1	2.835	4.471	3.252
ATOM	31	H	???	1	5.338	6.388	-4.982
ATOM	32	C	???	1	4.546	5.785	-5.447
ATOM	33	H	???	1	4.416	6.110	-6.502
ATOM	34	H	???	1	4.877	4.731	-5.444
ATOM	35	N	???	1	3.320	5.872	-4.685
ATOM	36	H	???	1	3.128	6.658	-4.056
ATOM	37	C	???	1	-2.520	2.757	3.431
ATOM	38	H	???	1	-3.108	2.846	4.359
ATOM	39	H	???	1	-1.779	3.574	3.402
ATOM	40	H	???	1	-3.187	2.796	2.549
ATOM	41	S	???	1	-1.546	1.214	3.401
ATOM	42	C	???	1	-2.868	-0.054	3.582
ATOM	43	H	???	1	-2.373	-0.986	3.275
ATOM	44	H	???	1	-3.629	0.179	2.816
ATOM	45	C	???	1	-3.536	-0.235	4.950
ATOM	46	H	???	1	-4.161	-1.146	4.873
ATOM	47	H	???	1	-4.221	0.606	5.162
ATOM	48	C	???	1	-2.607	-0.406	6.173
ATOM	49	N	???	1	-2.044	0.902	6.586
ATOM	50	H	???	1	-2.719	1.670	6.432
ATOM	51	H	???	1	-1.806	0.899	7.587
ATOM	52	H	???	1	-3.234	-0.827	6.976
ATOM	53	C	???	1	-1.473	-1.433	5.895
ATOM	54	O	???	1	-0.358	-0.960	5.468
ATOM	55	O	???	1	-1.717	-2.650	6.056
ATOM	56	S	???	1	-4.419	3.589	-8.182
ATOM	57	FE	???	1	-3.720	5.348	-6.967
ATOM	58	S	???	1	-4.006	5.494	-4.627
ATOM	59	FE	???	1	-5.990	5.284	-8.185
ATOM	60	S	???	1	-4.871	7.263	-7.490
ATOM	61	FE	???	1	-5.934	6.342	-5.758
ATOM	62	S	???	1	-7.476	5.032	-6.535
ATOM	63	FE	???	1	2.781	1.075	6.492
ATOM	64	S	???	1	-0.116	3.640	5.912
ATOM	65	FE	???	1	1.985	3.226	5.024
ATOM	66	S	???	1	2.019	0.995	4.364
ATOM	67	FE	???	1	0.019	1.190	5.720
ATOM	68	S	???	1	3.405	3.306	6.798
ATOM	69	FE	???	1	1.235	3.024	7.628
ATOM	70	S	???	1	0.965	0.717	7.908
ATOM	71	C	???	1	2.785	1.305	-3.076
ATOM	72	N	???	1	2.971	0.486	-4.135
ATOM	73	C	???	1	4.083	0.797	-4.816
ATOM	74	H	???	1	4.269	0.192	-5.718
ATOM	75	N	???	1	5.019	1.737	-4.550
ATOM	76	C	???	1	4.837	2.511	-3.447
ATOM	77	N	???	1	5.753	3.448	-3.094
ATOM	78	H	???	1	5.779	3.742	-2.108

```
ATOM    79  H   ???   1     6.626   3.494  -3.633
ATOM    80  C   ???   1     3.655   2.342  -2.690
ATOM    81  N   ???   1     3.159   3.043  -1.605
ATOM    82  C   ???   1     2.020   2.426  -1.327
ATOM    83  H   ???   1     1.313   2.708  -0.551
ATOM    84  N   ???   1     1.742   1.365  -2.166
ATOM    85  C   ???   1     0.542   0.519  -2.107
ATOM    86  O   ???   1    -0.580   1.372  -1.968
ATOM    87  H   ???   1     0.536  -0.038  -3.060
ATOM    88  C   ???   1     0.509  -0.491  -0.930
ATOM    89  O   ???   1     0.182  -1.755  -1.476
ATOM    90  H   ???   1     0.354  -2.416  -0.723
ATOM    91  H   ???   1     1.477  -0.518  -0.393
ATOM    92  C   ???   1    -0.648   0.041  -0.030
ATOM    93  O   ???   1    -1.405  -0.986   0.591
ATOM    94  H   ???   1    -0.982  -1.241   1.451
ATOM    95  H   ???   1    -0.270   0.768   0.716
ATOM    96  C   ???   1    -1.531   0.767  -1.055
ATOM    97  H   ???   1    -2.119  -0.001  -1.602
ATOM    98  C   ???   1    -2.444   1.839  -0.493
ATOM    99  H   ???   1    -3.112   1.377   0.258
ATOM   100  H   ???   1    -1.840   2.621   0.003
ATOM   101  O   ???   1     2.620   3.962  -5.646
ATOM   102  C   ???   1     2.413   4.868  -4.822
ATOM   103  C   ???   1     1.203   4.918  -3.915
ATOM   104  H   ???   1     0.846   5.963  -3.839
ATOM   105  H   ???   1     1.545   4.630  -2.901
ATOM   106  C   ???   1     0.083   4.002  -4.399
ATOM   107  H   ???   1     0.418   2.949  -4.321
ATOM   108  H   ???   1    -0.119   4.220  -5.469
ATOM   109  C   ???   1    -1.217   4.190  -3.621
ATOM   110  H   ???   1    -1.511   5.259  -3.648
ATOM   111  H   ???   1    -1.078   3.913  -2.557
ATOM   112  C   ???   1    -2.338   3.348  -4.237
ATOM   113  H   ???   1    -2.188   2.297  -3.922
ATOM   114  H   ???   1    -2.276   3.394  -5.342
ATOM   115  C   ???   1    -3.743   3.821  -3.853
ATOM   116  H   ???   1    -3.787   4.008  -2.763
ATOM   117  C   ???   1    -4.855   2.827  -4.241
ATOM   118  H   ???   1    -4.831   2.737  -5.353
ATOM   119  H   ???   1    -5.823   3.285  -3.971
ATOM   120  C   ???   1    -4.677   1.509  -3.551
ATOM   121  H   ???   1    -5.342   1.207  -2.730
ATOM   122  H   ???   1    -3.756   0.922  -3.700
ATOM   123  H   ???   1    -3.062   2.294  -1.289
END
```

IM2$_2$' (QM region of QM/MM calculation)

```
ATOM      1  H   ???     1      -4.423   8.940  -3.811
ATOM      2  C   ???     1      -5.279   9.267  -4.461
ATOM      3  H   ???     1      -4.905   9.405  -5.493
ATOM      4  H   ???     1      -5.655  10.235  -4.080
ATOM      5  S   ???     1      -6.713   8.104  -4.497
ATOM      6  H   ???     1      -0.244   7.484  -6.155
ATOM      7  C   ???     1      -1.252   7.029  -6.062
ATOM      8  H   ???     1      -2.022   7.817  -6.157
ATOM      9  H   ???     1      -1.342   6.576  -5.060
ATOM     10  S   ???     1      -1.471   5.737  -7.373
ATOM     11  H   ???     1      -8.122   7.588 -11.029
ATOM     12  C   ???     1      -7.738   7.171 -10.060
ATOM     13  H   ???     1      -8.577   6.995  -9.365
ATOM     14  H   ???     1      -7.051   7.910  -9.603
ATOM     15  S   ???     1      -6.832   5.586 -10.338
ATOM     16  H   ???     1       0.563   6.357  10.260
ATOM     17  C   ???     1       0.869   5.752   9.367
ATOM     18  H   ???     1       1.935   5.951   9.154
ATOM     19  H   ???     1       0.262   6.097   8.509
ATOM     20  S   ???     1       0.601   3.939   9.647
ATOM     21  H   ???     1       6.564   0.592   5.847
ATOM     22  C   ???     1       5.636   0.167   5.400
ATOM     23  H   ???     1       5.902  -0.654   4.706
ATOM     24  H   ???     1       5.118   0.956   4.827
ATOM     25  S   ???     1       4.507  -0.466   6.726
ATOM     26  H   ???     1       5.102   3.980   2.380
ATOM     27  C   ???     1       4.431   3.541   3.157
ATOM     28  H   ???     1       4.940   3.566   4.138
ATOM     29  H   ???     1       4.238   2.488   2.883
ATOM     30  S   ???     1       2.824   4.460   3.245
ATOM     31  H   ???     1       5.326   6.385  -4.976
ATOM     32  C   ???     1       4.519   5.778  -5.429
ATOM     33  H   ???     1       4.374   6.108  -6.480
ATOM     34  H   ???     1       4.856   4.727  -5.437
ATOM     35  N   ???     1       3.293   5.849  -4.655
ATOM     36  H   ???     1       3.096   6.633  -4.025
ATOM     37  C   ???     1      -2.460   2.772   3.446
ATOM     38  H   ???     1      -3.041   2.896   4.375
ATOM     39  H   ???     1      -1.723   3.589   3.375
ATOM     40  H   ???     1      -3.134   2.772   2.570
ATOM     41  S   ???     1      -1.483   1.230   3.465
ATOM     42  C   ???     1      -2.824  -0.023   3.616
ATOM     43  H   ???     1      -2.341  -0.962   3.310
ATOM     44  H   ???     1      -3.575   0.224   2.844
ATOM     45  C   ???     1      -3.502  -0.198   4.979
ATOM     46  H   ???     1      -4.126  -1.111   4.907
ATOM     47  H   ???     1      -4.188   0.644   5.186
ATOM     48  C   ???     1      -2.566  -0.362   6.197
ATOM     49  N   ???     1      -1.984   0.944   6.590
ATOM     50  H   ???     1      -2.651   1.716   6.426
```

ATOM	51	H	???	1	-1.752	0.952	7.593
ATOM	52	H	???	1	-3.187	-0.774	7.011
ATOM	53	C	???	1	-1.440	-1.390	5.907
ATOM	54	O	???	1	-0.329	-0.908	5.478
ATOM	55	O	???	1	-1.685	-2.608	6.054
ATOM	56	S	???	1	-4.393	3.640	-8.269
ATOM	57	FE	???	1	-3.723	5.336	-6.957
ATOM	58	S	???	1	-4.037	5.475	-4.625
ATOM	59	FE	???	1	-5.974	5.321	-8.211
ATOM	60	S	???	1	-4.842	7.274	-7.464
ATOM	61	FE	???	1	-5.952	6.346	-5.765
ATOM	62	S	???	1	-7.518	5.093	-6.606
ATOM	63	FE	???	1	2.750	1.030	6.476
ATOM	64	S	???	1	-0.154	3.612	5.908
ATOM	65	FE	???	1	1.942	3.202	5.003
ATOM	66	S	???	1	1.991	0.968	4.353
ATOM	67	FE	???	1	0.044	1.197	5.740
ATOM	68	S	???	1	3.358	3.277	6.785
ATOM	69	FE	???	1	1.184	2.996	7.621
ATOM	70	S	???	1	0.950	0.696	7.912
ATOM	71	C	???	1	2.784	1.306	-3.071
ATOM	72	N	???	1	2.967	0.486	-4.130
ATOM	73	C	???	1	4.079	0.796	-4.814
ATOM	74	H	???	1	4.262	0.190	-5.715
ATOM	75	N	???	1	5.016	1.736	-4.551
ATOM	76	C	???	1	4.837	2.511	-3.448
ATOM	77	N	???	1	5.755	3.445	-3.095
ATOM	78	H	???	1	5.780	3.742	-2.110
ATOM	79	H	???	1	6.626	3.491	-3.635
ATOM	80	C	???	1	3.654	2.344	-2.690
ATOM	81	N	???	1	3.161	3.049	-1.606
ATOM	82	C	???	1	2.023	2.433	-1.324
ATOM	83	H	???	1	1.317	2.718	-0.548
ATOM	84	N	???	1	1.743	1.369	-2.159
ATOM	85	C	???	1	0.541	0.526	-2.093
ATOM	86	O	???	1	-0.576	1.384	-1.941
ATOM	87	H	???	1	0.524	-0.027	-3.049
ATOM	88	C	???	1	0.512	-0.490	-0.921
ATOM	89	O	???	1	0.182	-1.750	-1.471
ATOM	90	H	???	1	0.355	-2.415	-0.720
ATOM	91	H	???	1	1.483	-0.519	-0.388
ATOM	92	C	???	1	-0.641	0.040	-0.012
ATOM	93	O	???	1	-1.402	-0.988	0.602
ATOM	94	H	???	1	-0.981	-1.248	1.462
ATOM	95	H	???	1	-0.257	0.759	0.738
ATOM	96	C	???	1	-1.524	0.775	-1.029
ATOM	97	H	???	1	-2.116	0.012	-1.578
ATOM	98	C	???	1	-2.431	1.845	-0.452
ATOM	99	H	???	1	-3.104	1.377	0.291
ATOM	100	H	???	1	-1.823	2.615	0.057

ATOM	101	O	???	1	2.592	3.967	-5.665
ATOM	102	C	???	1	2.374	4.856	-4.826
ATOM	103	C	???	1	1.141	4.887	-3.940
ATOM	104	H	???	1	0.767	5.928	-3.874
ATOM	105	H	???	1	1.473	4.615	-2.918
ATOM	106	C	???	1	0.030	3.946	-4.422
ATOM	107	H	???	1	0.386	2.901	-4.337
ATOM	108	H	???	1	-0.181	4.159	-5.490
ATOM	109	C	???	1	-1.270	4.120	-3.629
ATOM	110	H	???	1	-1.569	5.187	-3.665
ATOM	111	H	???	1	-1.104	3.862	-2.564
ATOM	112	C	???	1	-2.437	3.281	-4.183
ATOM	113	H	???	1	-2.335	2.232	-3.831
ATOM	114	H	???	1	-2.412	3.278	-5.291
ATOM	115	C	???	1	-3.800	3.845	-3.751
ATOM	116	H	???	1	-3.733	4.122	-2.682
ATOM	117	C	???	1	-5.001	2.887	-3.924
ATOM	118	H	???	1	-5.909	3.404	-3.558
ATOM	119	H	???	1	-4.821	2.024	-3.245
ATOM	120	C	???	1	-5.231	2.429	-5.324
ATOM	121	H	???	1	-4.705	1.554	-5.729
ATOM	122	H	???	1	-5.827	3.031	-6.021
ATOM	123	H	???	1	-3.044	2.322	-1.238

END

$PS1_2$ (QM region of QM/MM calculation)

ATOM	1	H	???	1	-4.420	8.951	-3.809
ATOM	2	C	???	1	-5.271	9.298	-4.455
ATOM	3	H	???	1	-4.906	9.404	-5.494
ATOM	4	H	???	1	-5.607	10.288	-4.092
ATOM	5	S	???	1	-6.744	8.187	-4.445
ATOM	6	H	???	1	-0.244	7.490	-6.157
ATOM	7	C	???	1	-1.256	7.047	-6.067
ATOM	8	H	???	1	-2.009	7.856	-6.131
ATOM	9	H	???	1	-1.342	6.571	-5.076
ATOM	10	S	???	1	-1.520	5.787	-7.405
ATOM	11	H	???	1	-8.120	7.585	-11.031
ATOM	12	C	???	1	-7.734	7.167	-10.063
ATOM	13	H	???	1	-8.567	7.035	-9.352
ATOM	14	H	???	1	-7.009	7.882	-9.628
ATOM	15	S	???	1	-6.903	5.526	-10.297
ATOM	16	H	???	1	0.563	6.356	10.261
ATOM	17	C	???	1	0.868	5.749	9.368
ATOM	18	H	???	1	1.934	5.948	9.155
ATOM	19	H	???	1	0.262	6.094	8.510
ATOM	20	S	???	1	0.600	3.936	9.649
ATOM	21	H	???	1	6.565	0.591	5.847
ATOM	22	C	???	1	5.638	0.163	5.402

ATOM	23	H	???	1	5.907	-0.657	4.708
ATOM	24	H	???	1	5.117	0.951	4.829
ATOM	25	S	???	1	4.510	-0.470	6.730
ATOM	26	H	???	1	5.101	3.981	2.379
ATOM	27	C	???	1	4.427	3.544	3.155
ATOM	28	H	???	1	4.930	3.573	4.139
ATOM	29	H	???	1	4.236	2.489	2.886
ATOM	30	S	???	1	2.818	4.463	3.230
ATOM	31	H	???	1	5.312	6.380	-4.969
ATOM	32	C	???	1	4.487	5.770	-5.404
ATOM	33	H	???	1	4.328	6.099	-6.453
ATOM	34	H	???	1	4.826	4.719	-5.418
ATOM	35	N	???	1	3.258	5.835	-4.622
ATOM	36	H	???	1	3.068	6.614	-3.982
ATOM	37	C	???	1	-2.453	2.748	3.418
ATOM	38	H	???	1	-3.062	2.862	4.331
ATOM	39	H	???	1	-1.711	3.564	3.386
ATOM	40	H	???	1	-3.101	2.765	2.522
ATOM	41	S	???	1	-1.481	1.203	3.451
ATOM	42	C	???	1	-2.830	-0.041	3.613
ATOM	43	H	???	1	-2.354	-0.986	3.315
ATOM	44	H	???	1	-3.582	0.205	2.842
ATOM	45	C	???	1	-3.503	-0.203	4.980
ATOM	46	H	???	1	-4.135	-1.111	4.915
ATOM	47	H	???	1	-4.182	0.646	5.185
ATOM	48	C	???	1	-2.565	-0.367	6.196
ATOM	49	N	???	1	-1.976	0.937	6.584
ATOM	50	H	???	1	-2.637	1.714	6.417
ATOM	51	H	???	1	-1.739	0.947	7.585
ATOM	52	H	???	1	-3.185	-0.775	7.012
ATOM	53	C	???	1	-1.442	-1.400	5.906
ATOM	54	O	???	1	-0.331	-0.923	5.478
ATOM	55	O	???	1	-1.694	-2.618	6.053
ATOM	56	S	???	1	-4.613	3.566	-7.627
ATOM	57	FE	???	1	-3.803	5.655	-6.912
ATOM	58	S	???	1	-4.120	5.634	-4.624
ATOM	59	FE	???	1	-6.169	5.369	-8.100
ATOM	60	S	???	1	-5.106	7.372	-7.568
ATOM	61	FE	???	1	-6.096	6.449	-5.734
ATOM	62	S	???	1	-7.699	5.157	-6.520
ATOM	63	FE	???	1	2.762	1.034	6.483
ATOM	64	S	???	1	-0.161	3.604	5.902
ATOM	65	FE	???	1	1.936	3.193	4.997
ATOM	66	S	???	1	1.998	0.954	4.349
ATOM	67	FE	???	1	0.062	1.188	5.747
ATOM	68	S	???	1	3.359	3.288	6.776
ATOM	69	FE	???	1	1.187	2.997	7.620
ATOM	70	S	???	1	0.953	0.692	7.912
ATOM	71	C	???	1	2.782	1.316	-3.086
ATOM	72	N	???	1	2.969	0.496	-4.145

ATOM	73	C	???	1	4.083	0.808	-4.825
ATOM	74	H	???	1	4.270	0.203	-5.725
ATOM	75	N	???	1	5.018	1.749	-4.558
ATOM	76	C	???	1	4.835	2.521	-3.453
ATOM	77	N	???	1	5.755	3.451	-3.092
ATOM	78	H	???	1	5.778	3.743	-2.105
ATOM	79	H	???	1	6.630	3.494	-3.628
ATOM	80	C	???	1	3.649	2.355	-2.701
ATOM	81	N	???	1	3.153	3.054	-1.616
ATOM	82	C	???	1	2.016	2.434	-1.335
ATOM	83	H	???	1	1.315	2.711	-0.552
ATOM	84	N	???	1	1.739	1.373	-2.175
ATOM	85	C	???	1	0.547	0.518	-2.118
ATOM	86	O	???	1	-0.587	1.361	-2.008
ATOM	87	H	???	1	0.558	-0.054	-3.062
ATOM	88	C	???	1	0.506	-0.477	-0.928
ATOM	89	O	???	1	0.184	-1.747	-1.465
ATOM	90	H	???	1	0.359	-2.404	-0.706
ATOM	91	H	???	1	1.471	-0.498	-0.384
ATOM	92	C	???	1	-0.659	0.064	-0.043
ATOM	93	O	???	1	-1.412	-0.960	0.587
ATOM	94	H	???	1	-0.995	-1.194	1.455
ATOM	95	H	???	1	-0.290	0.803	0.695
ATOM	96	C	???	1	-1.538	0.765	-1.088
ATOM	97	H	???	1	-2.114	-0.020	-1.623
ATOM	98	C	???	1	-2.464	1.843	-0.556
ATOM	99	H	???	1	-3.150	1.391	0.184
ATOM	100	H	???	1	-1.871	2.629	-0.055
ATOM	101	O	???	1	2.538	3.987	-5.677
ATOM	102	C	???	1	2.320	4.861	-4.821
ATOM	103	C	???	1	1.060	4.887	-3.956
ATOM	104	H	???	1	0.707	5.933	-3.864
ATOM	105	H	???	1	1.365	4.576	-2.936
ATOM	106	C	???	1	-0.062	3.977	-4.493
ATOM	107	H	???	1	0.275	2.926	-4.411
ATOM	108	H	???	1	-0.218	4.210	-5.567
ATOM	109	C	???	1	-1.415	4.137	-3.769
ATOM	110	H	???	1	-1.697	5.209	-3.759
ATOM	111	H	???	1	-1.319	3.815	-2.714
ATOM	112	C	???	1	-2.555	3.348	-4.461
ATOM	113	H	???	1	-2.476	2.280	-4.164
ATOM	114	H	???	1	-2.437	3.431	-5.558
ATOM	115	C	???	1	-3.972	3.855	-4.134
ATOM	116	H	???	1	-4.097	3.871	-3.034
ATOM	117	C	???	1	-5.184	3.081	-4.680
ATOM	118	H	???	1	-6.071	3.689	-4.419
ATOM	119	H	???	1	-5.268	2.145	-4.087
ATOM	120	C	???	1	-5.344	2.655	-6.165
ATOM	121	H	???	1	-5.007	1.608	-6.280
ATOM	122	H	???	1	-6.420	2.704	-6.398

```
ATOM    123  H   ???    1      -3.061   2.298  -1.369
END
```

PS2$_2$ (QM region of QM/MM calculation)

```
ATOM      1  H   ???    1      -4.428   8.934  -3.812
ATOM      2  C   ???    1      -5.291   9.246  -4.463
ATOM      3  H   ???    1      -4.924   9.378  -5.498
ATOM      4  H   ???    1      -5.674  10.214  -4.090
ATOM      5  S   ???    1      -6.711   8.055  -4.470
ATOM      6  H   ???    1      -0.234   7.486  -6.157
ATOM      7  C   ???    1      -1.232   7.028  -6.070
ATOM      8  H   ???    1      -2.006   7.815  -6.141
ATOM      9  H   ???    1      -1.317   6.546  -5.081
ATOM     10  S   ???    1      -1.424   5.770  -7.420
ATOM     11  H   ???    1      -8.120   7.587 -11.023
ATOM     12  C   ???    1      -7.735   7.175 -10.046
ATOM     13  H   ???    1      -8.569   7.117  -9.327
ATOM     14  H   ???    1      -6.972   7.870  -9.643
ATOM     15  S   ???    1      -6.993   5.494 -10.224
ATOM     16  H   ???    1       0.563   6.356  10.261
ATOM     17  C   ???    1       0.869   5.751   9.367
ATOM     18  H   ???    1       1.934   5.950   9.154
ATOM     19  H   ???    1       0.262   6.095   8.510
ATOM     20  S   ???    1       0.601   3.938   9.648
ATOM     21  H   ???    1       6.564   0.592   5.847
ATOM     22  C   ???    1       5.636   0.166   5.401
ATOM     23  H   ???    1       5.903  -0.653   4.705
ATOM     24  H   ???    1       5.116   0.956   4.830
ATOM     25  S   ???    1       4.509  -0.470   6.727
ATOM     26  H   ???    1       5.102   3.980   2.380
ATOM     27  C   ???    1       4.431   3.541   3.156
ATOM     28  H   ???    1       4.938   3.567   4.138
ATOM     29  H   ???    1       4.238   2.487   2.884
ATOM     30  S   ???    1       2.822   4.460   3.242
ATOM     31  H   ???    1       5.319   6.383  -4.973
ATOM     32  C   ???    1       4.504   5.775  -5.419
ATOM     33  H   ???    1       4.352   6.107  -6.468
ATOM     34  H   ???    1       4.844   4.724  -5.432
ATOM     35  N   ???    1       3.277   5.838  -4.640
ATOM     36  H   ???    1       3.076   6.622  -4.011
ATOM     37  C   ???    1      -2.459   2.771   3.443
ATOM     38  H   ???    1      -3.043   2.893   4.371
ATOM     39  H   ???    1      -1.721   3.589   3.380
ATOM     40  H   ???    1      -3.129   2.776   2.564
ATOM     41  S   ???    1      -1.482   1.229   3.462
ATOM     42  C   ???    1      -2.824  -0.023   3.615
ATOM     43  H   ???    1      -2.341  -0.963   3.312
ATOM     44  H   ???    1      -3.575   0.223   2.843
```

ATOM	45	C	???	1	-3.501	-0.197	4.979
ATOM	46	H	???	1	-4.127	-1.109	4.908
ATOM	47	H	???	1	-4.186	0.647	5.185
ATOM	48	C	???	1	-2.565	-0.361	6.197
ATOM	49	N	???	1	-1.982	0.944	6.589
ATOM	50	H	???	1	-2.647	1.717	6.425
ATOM	51	H	???	1	-1.746	0.952	7.591
ATOM	52	H	???	1	-3.187	-0.772	7.011
ATOM	53	C	???	1	-1.440	-1.391	5.906
ATOM	54	O	???	1	-0.330	-0.910	5.476
ATOM	55	O	???	1	-1.687	-2.609	6.055
ATOM	56	S	???	1	-4.283	3.662	-8.352
ATOM	57	FE	???	1	-3.676	5.441	-7.038
ATOM	58	S	???	1	-3.905	5.575	-4.668
ATOM	59	FE	???	1	-5.960	5.162	-8.142
ATOM	60	S	???	1	-5.053	7.235	-7.573
ATOM	61	FE	???	1	-5.801	6.332	-5.684
ATOM	62	S	???	1	-6.822	4.294	-6.064
ATOM	63	FE	???	1	2.750	1.023	6.480
ATOM	64	S	???	1	-0.151	3.613	5.909
ATOM	65	FE	???	1	1.945	3.197	5.004
ATOM	66	S	???	1	1.993	0.961	4.352
ATOM	67	FE	???	1	0.050	1.198	5.741
ATOM	68	S	???	1	3.363	3.271	6.785
ATOM	69	FE	???	1	1.188	2.994	7.623
ATOM	70	S	???	1	0.947	0.693	7.913
ATOM	71	C	???	1	2.784	1.306	-3.074
ATOM	72	N	???	1	2.968	0.486	-4.132
ATOM	73	C	???	1	4.079	0.797	-4.816
ATOM	74	H	???	1	4.262	0.191	-5.718
ATOM	75	N	???	1	5.016	1.737	-4.552
ATOM	76	C	???	1	4.836	2.511	-3.449
ATOM	77	N	???	1	5.755	3.443	-3.094
ATOM	78	H	???	1	5.779	3.741	-2.109
ATOM	79	H	???	1	6.627	3.490	-3.634
ATOM	80	C	???	1	3.653	2.345	-2.692
ATOM	81	N	???	1	3.159	3.047	-1.608
ATOM	82	C	???	1	2.023	2.430	-1.325
ATOM	83	H	???	1	1.318	2.712	-0.546
ATOM	84	N	???	1	1.743	1.366	-2.161
ATOM	85	C	???	1	0.544	0.520	-2.097
ATOM	86	O	???	1	-0.578	1.372	-1.956
ATOM	87	H	???	1	0.535	-0.038	-3.050
ATOM	88	C	???	1	0.511	-0.491	-0.921
ATOM	89	O	???	1	0.179	-1.753	-1.469
ATOM	90	H	???	1	0.354	-2.416	-0.716
ATOM	91	H	???	1	1.480	-0.521	-0.385
ATOM	92	C	???	1	-0.644	0.042	-0.017
ATOM	93	O	???	1	-1.404	-0.984	0.601
ATOM	94	H	???	1	-0.984	-1.239	1.463

```
ATOM     95  H   ???     1      -0.263   0.766   0.730
ATOM     96  C   ???     1      -1.526   0.769  -1.041
ATOM     97  H   ???     1      -2.116  -0.000  -1.585
ATOM     98  C   ???     1      -2.437   1.843  -0.476
ATOM     99  H   ???     1      -3.113   1.380   0.267
ATOM    100  H   ???     1      -1.831   2.616   0.030
ATOM    101  O   ???     1       2.581   3.964  -5.669
ATOM    102  C   ???     1       2.355   4.847  -4.825
ATOM    103  C   ???     1       1.108   4.867  -3.951
ATOM    104  H   ???     1       0.731   5.906  -3.881
ATOM    105  H   ???     1       1.430   4.587  -2.928
ATOM    106  C   ???     1       0.001   3.924  -4.450
ATOM    107  H   ???     1       0.369   2.882  -4.383
ATOM    108  H   ???     1      -0.211   4.156  -5.514
ATOM    109  C   ???     1      -1.303   4.068  -3.650
ATOM    110  H   ???     1      -1.586   5.140  -3.637
ATOM    111  H   ???     1      -1.135   3.762  -2.599
ATOM    112  C   ???     1      -2.499   3.274  -4.229
ATOM    113  H   ???     1      -2.453   2.224  -3.871
ATOM    114  H   ???     1      -2.447   3.274  -5.336
ATOM    115  C   ???     1      -3.837   3.924  -3.816
ATOM    116  H   ???     1      -3.758   4.166  -2.740
ATOM    117  C   ???     1      -5.134   3.124  -4.031
ATOM    118  H   ???     1      -5.946   3.576  -3.432
ATOM    119  H   ???     1      -4.983   2.104  -3.616
ATOM    120  C   ???     1      -5.565   3.024  -5.501
ATOM    121  H   ???     1      -4.703   3.121  -6.183
ATOM    122  H   ???     1      -6.005   2.042  -5.736
ATOM    123  H   ???     1      -3.046   2.316  -1.269
END
```
